# Detecting temporal protein complexes from dynamic protein-protein interaction networks

**DOI:** 10.1186/1471-2105-15-335

**Published:** 2014-10-04

**Authors:** Le Ou-Yang, Dao-Qing Dai, Xiao-Li Li, Min Wu, Xiao-Fei Zhang, Peng Yang

**Affiliations:** Intelligent Data Center and Department of Mathematics, Sun Yat-Sen University, Guangzhou, 510275 China; Institute for Infocomm Research (I2R), A*STAR, 1 Fusionopolis Way, Singapore, 138632 Singapore; School of Mathematics and Statistics, Central China Normal University, Wuhan, 430079 China

**Keywords:** Dynamic protein-protein interaction, Gene expression, Stable interaction, Transient interaction, Protein complex

## Abstract

**Background:**

Proteins dynamically interact with each other to perform their biological functions. The dynamic operations of protein interaction networks (PPI) are also reflected in the dynamic formations of protein complexes. Existing protein complex detection algorithms usually overlook the inherent temporal nature of protein interactions within PPI networks. Systematically analyzing the temporal protein complexes can not only improve the accuracy of protein complex detection, but also strengthen our biological knowledge on the dynamic protein assembly processes for cellular organization.

**Results:**

In this study, we propose a novel computational method to predict temporal protein complexes. Particularly, we first construct a series of dynamic PPI networks by joint analysis of time-course gene expression data and protein interaction data. Then a Time Smooth Overlapping Complex Detection model (TS-OCD) has been proposed to detect temporal protein complexes from these dynamic PPI networks. TS-OCD can naturally capture the smoothness of networks between consecutive time points and detect overlapping protein complexes at each time point. Finally, a nonnegative matrix factorization based algorithm is introduced to merge those very similar temporal complexes across different time points.

**Conclusions:**

Extensive experimental results demonstrate the proposed method is very effective in detecting temporal protein complexes than the state-of-the-art complex detection techniques.

**Electronic supplementary material:**

The online version of this article (doi:10.1186/1471-2105-15-335) contains supplementary material, which is available to authorized users.

## Background

With the technological advances in high-throughput screening techniques, large-scale protein-protein interaction (PPI) data have been generated and catalogued for many species [1–3]. Proteins seldom act alone, and they often bind together to form complexes to carry out their biological functions [4–6]. Comprehensive investigation of protein complexes could help to reveal the structure of PPI networks, predict protein functions and elucidate cellular mechanisms underlying various diseases [7]. Computational detection of complexes has thus attracted tremendous attentions during the past decade [6, 8–14].

According to their life time, PPIs could be classified into stable or transient PPIs [15, 16]. Stable PPIs which are important in maintaining the cell fitness and stability are usually permanent and irreversible. Meanwhile, transient PPIs can associate and dissociate temporarily, and thus they provide a mechanism for the cell to quickly respond to extracellular stimuli. As physical interactions determined by popular high-throughput technologies, e.g. yeast two-hybrid (Y2H) and Tandem Affinity Purification with mass spectrometry (TAP-MS) lack of temporal information, majority of existing complex detection methods treat the PPI network as a *static network* that can not be used to detect *temporal* protein complexes. In reality, however, cellular systems are highly dynamic and responsive to environmental cues [17]. The real PPI network in cell keeps changing over different stages of the cell cycle [18], leading to *multiple dynamic protein interaction networks*. As such, it is desirable to design novel computational methods that can take the inherent dynamic characteristics of PPI networks into consideration to better detect *temporal* protein complexes.

Nevertheless, the advent of DNA microarray technologies has enabled the differential expressions of thousands of genes under various experimental conditions to be monitored simultaneously and quantitatively [19, 20], which provides the useful *temporal* information to complement the *static* protein interaction data in the gene level. There have been some attempts to investigate the temporal properties for individual proteins and protein interactions by integrating PPI data with time-course gene expression data [21–29]. For example, in [22], the authors proposed a three-sigma principle to identify *active* time points for individual proteins. They further investigated the temporal protein associations and protein state transition on the identified active time points.

Temporal protein complexes are typically constructed by the dynamic assembly or disassembly of proteins to perform various biological functions. Tracking the temporal protein complexes could reveal important insights into dynamic modular mechanisms and improve our understanding on the disease pathways etc [23, 30]. To detect temporal protein complexes, we need to leverage the temporal information from gene expression data to construct time-evolving dynamic protein interaction networks. In [31], the authors incorporated the "time" factor for proteins in the form of cell-cycle phases into the analysis of complexes and studied the temporal phenomena of complex assembly and disassembly across various cell cycles. Wang *et al.* identified temporal protein complexes from the dynamic PPI networks by applying *static* complex detection methods (e.g., MCL) for each time point [22]. In [28], the authors proposed DHAC (Dynamical Hierarchical Agglomerative Clustering) complex mining method, to detect temporal complexes from individual dynamic PPI networks.

We observe that the above few methods for predicting temporal protein complexes suffer from the following two major limitations. Firstly, their methods just focus on the individual dynamic PPI networks and fully ignore the correlations between the networks at consecutive time points. Note that while there are different temporal complexes occur at different time points, many protein complexes will still form stable macromolecular complexes to perform their important biological functions [21]. As many *stable* interactions that perform fundamental roles for the cell are conserved across different time points, the corresponding complexes will also occur in multiple consecutive dynamics PPI networks and they should thus change smoothly across time [24, 26], to maintain the cell fitness and stability as well as to avoid the adverse disruption of the basic operations of the cell. These existing methods, however, have overlooked the smoothness of the temporal complexes at different time points and simply apply *static* complex detection methods for each individual dynamic PPI network. Secondly, as multi-functional proteins are often involved in different complexes, it is highly desirable to discover overlapping complexes to better decipher the inherent overlapping modular structures of PPI networks. However, existing methods, namely DHAC and MCL, do not generate the overlapping protein complexes and they are thus less accurate.

To address the above two issues, in this paper we propose a novel technique to detect temporal protein complexes from the dynamic PPI networks. We first construct a series of dynamic PPI networks by detecting *stable* interactions and *transient* interactions by integrating protein interaction data and gene expression data. Particularly, the stable interactions are reserved across different time points to serve as the backbone of the protein interaction networks, while the existence of a transient interaction at a certain time point depends on the specific activities and functions required from the two associated proteins. Then, based on the concept of overlapping temporal communities [32], we propose a novel Time Smooth Overlapping Complex Detection model (TS-OCD) to detect overlapping temporal protein complexes from the constructed dynamic PPI networks, which allows individual complex to grow and shrink across different time points. Finally, a Nonnegative Matrix Factorization (NMF) based method is introduced to effectively merge those very similar temporal complexes across time and track their evolutionary process. We have performed extensive experiments to evaluate the performance of our TS-OCD model. Experimental results show that TS-OCD is able to achieve significantly better results than the state-of-the-art algorithms for detecting protein complexes. Moreover, our algorithm is accessible as a tool, which could be downloaded from http://mail.sysu.edu.cn/home/stsddq@mail.sysu.edu.cn/dai/others/TSOCD.zip.

## Methods

In this section, we first present how to construct dynamic PPI networks, and subsequently introduce how to detect overlapping temporal protein complexes from the constructed dynamic PPI networks.

### Constructing dynamic PPI networks

The dynamic protein-protein interaction networks (DPPI networks) are constructed by integrating time-course gene expression data with static PPI networks. A static PPI network is often modelled as an undirected graph *G* = (*V*,*E*), where *V* consists of |*V*| = *N* proteins and *E* consists of |*E*| edges (protein interactions under different conditions between two proteins in *V*). The time-course gene expression data of these *N* proteins across *T* time points are represented by a *N* × *T* matrix *GE*, which represents the expression level of *N* genes across *T* time points.

Now, we infer a DPPI network for each time point from *GE* and *G*. Existing methods construct DPPI networks solely by determining the peak time points of expression for each protein [22] and the connections among the networks at different time points are ignored. To address this problem, we first extract *stable* protein interactions from *G*, which are supposed to appear at all time points, as they are encoded by globally co-expressed gene pairs [27]. Particularly, for each protein interaction in *G*, we calculate their Pearson Correlation Coefficient (PCC) based on their gene expression profiles across all time points in *GE*. Then the protein interactions with PCC values greater than a certain cutoff *δ* are defined as stable interactions due to their corresponding globally co-expressed genes (we will discuss how to determine the value of *δ* in next section). These stable interactions represent the static part of the DPPI networks and are likely to be reserved across all time points. Note a *N* × *N* symmetric matrix *S* is introduced to indicate the stable interactions in the given PPI network *G* = (*V*,*E*), where *S*_*ij*_ = 1 if protein *i* and *j* has a stable interaction, i.e. *e*_*ij*_ ∈ *E* and *PCC*(*e*_*ij*_) > *δ*; *S*_*ij*_ = 0 otherwise.

The *dynamic* parts of the DPPI network for each time point *t*(1 ≤ *t* ≤ *T*) are inferred from *GE* and *G*, as a transient interaction only presents at certain time points when both of the associated proteins are in their active forms. Particularly, at time point *t*, a protein *i* is considered to be in its active form if its expression value is above or equal to its active threshold which could be denoted as *AT*(*i*), as discussed in [22]. The active threshold for each protein is determined as follows:
1

where  and *σ*(*i*) are the algorithm mean and standard deviation of the expression values over times 1 to *T* for protein *i* respectively, and *F*(*i*) = 1/(1 + *σ*^2^(*i*)) is a weight function which reflects the fluctuation of the expression values of protein *i*. For more details, please refer to [22]. For each edge in the static PPI network (i.e., *e*_*ij*_ ∈ *E*), it is presented at time point *t* if proteins *i* and *j* are in their active states (i.e., *GE*_*it*_ ≥ *AT*(*i*) and *GE*_*jt*_ ≥ *AT*(*j*)). The dynamic PPI networks can be represented by a set of graphs, *G*^(*t*)^ = (*V*,*E*^(*t*)^), *t* = 1,…,*T*, where *V* denotes the original set of proteins and *E*^(*t*)^ represents the set of edges presented at time point *t*. Particularly, edge  if *S*_*ij*_ = 1 (i.e. stable interaction) or *e*_*ij*_ ∈ *E*, *GE*_*it*_ ≥ *AT*(*i*) and *GE*_*jt*_ ≥ *AT*(*j*) (i.e. transient interaction). For each dynamic PPI network *G*^(*t*)^,  is introduced to represent its adjacency matrix, where  if  and  otherwise.

### Detecting overlapping temporal protein complexes

Our objective is to infer *D*^(*t*)^(1 ≤ *t* ≤ *T*), a sequence of time-evolving protein complexes, from the dynamic networks *G*^(*t*)^(1 ≤ *t* ≤ *T*). Let  contains *r*_*t*_ predicted complexes at time point *t*. We define a *N* × *r*_*t*_ protein-complex assignment matrix *H*^(*t*)^ to indicate the membership of proteins in complexes, where  if protein *i* belongs to a complex , and  otherwise. Here we allow *overlapping* proteins occur in multiple protein complexes simultaneously, i.e. , , and *k* ≠ *z*. Obviously, if we can compute *H*^(*t*)^, we can easily infer *D*^(*t*)^.

We further introduce another *N* × *N* matrix *U*^(*t*)^, where each element  is the number of predicted complexes in *D*^(*t*)^ which contain both proteins *i* and *j*, i.e., . Clearly, *U*^(*t*)^ represents the co-complex membership among proteins at the time point *t*, which allows a protein to belong to more than one complex. Meanwhile, we have .

#### Model formulation

In order to predict *D*^(*t*)^, we first infer *U*^(*t*)^ from the dynamic networks *G*^(*t*)^,1 ≤ *t* ≤ *T*. Particularly, We study the following three factors that are relevant for estimating *U*^(*t*)^.

Firstly, based on the assumption that proteins belong to same complexes tend to interact with each other,  represents the expected number of interactions in complex *k* that lie between proteins *i* and *j* at time point *t*. Considering all complexes at time point *t*,  represents the expected total number of interactions between protein *i* and *j* in terms of all the *r*_*t*_ complexes. Similarly to [13, 33], we assume the observed interaction between protein *i* and *j* at time point *t* is independently generated by a Poisson distribution with mean . Given the generative model, we can estimate *U*^(*t*)^ from *A*^(*t*)^ by maximizing the following likelihood function:
2

Taking the negative logarithm and dropping constants, maximizing the above likelihood function is equal to minimizing the following loss function:
3

Secondly, stable interactions are preserved across all the dynamic PPI networks, whereas the transient interactions only present at some special time points and absent at the other time points. Therefore, we introduce a smoothness regularization term *R* to enforce the stable interactions (with *S*_*ij*_ = 1) and their corresponding complex membership  in *U*^(*t*)^ to change smoothly over time, rather than change dramatically between two consecutive time points. Here, the smooth regularization term  shows the temporal smoothness between  and . Correspondingly,  measures the overall smoothness across all time points.

Finally, as , the rank of matrix *U*^(*t*)^ cannot be larger than the number of complexes *r*_*t*_. As we have no prior knowledge on *r*_*t*_, a low rank restriction for each *U*^(*t*)^ is thus needed during estimating *U*^(*t*)^. In this paper, we use the trace norm constraint ∥*U*^(*t*)^∥_∗_ as a relaxation of the low rank constraint [32], which prevents our model from producing too many complexes and controls the overlaps among complexes. In particular, ∥*U*^(*t*)^∥_∗_ is the sum of singular values of *U*^(*t*)^. According to the definition, it is easy to obtain , where ∥·∥_*F*_ denotes Frobenius norm.

#### Temporal protein complex detection

Taking into account all the above three factors and dropping those constants, our objective function, aiming to minimize the loss function, the regularization term for smoothness, as well as the low rank constraint, is defined as follows:
4

where *λ* ≥ 0 and *β* ≥ 0 are the tradeoff parameters that control the balance among the three factors. The optimization problem (4) is combinatorial as *U*^(*t*)^ specifies all the possible co-complex memberships among proteins at the time point *t*. As such, exhaustive search is impractical since there are exponentially many possible combinations. To address this problem, we relax the constrains of *U*^(*t*)^ and *H*^(*t*)^ from integers ( ) to real numbers with *U*^(*t*)^ ≥ 0 and *H*^(*t*)^ ≥ 0.

Ideally, we could first compute the optimal solution  and then extract a set of predicted complexes from it easily. However, because of the real number relaxation, while  could approximate the underlying complex structure of *A*^(*t*)^, it may not have a clear block structures that can clearly indicate protein complexes where protein pairs inside the complexes all have high  values. Therefore, we still need to extract clusters from  via clustering methods such as spectral clustering. In this paper, instead of taking two steps to infer *H*^(*t*)^, we present a novel Time Smooth Overlapping Complex Detection (TS-OCD) model with the following objective function by substituting  into (4):
5

Therefore, we could *directly* extract clusters from the optimal solution . To solve the above objective function (5), we adopt the multiplicative update rules [34] which are special cases of gradient descend method with an automatic step parameter selection and could naturally keep the nonnegativity of *H*^(*t*)^. Please refer to the Additional file 1 for more details. Note each element  of  is a continuous value, describing the propensity of protein *i* belonging to a predicted complex *k*. We discretize  into the final protein-complex assignment matrix *H*^(*t*)^^⋆^ with the rules in Equation (6). Particularly, we assign protein *i* to the predicted complex *k* if the value of  exceed a threshold *τ*.
6

Here,  represents protein *i* is in predicted complex *k* at time point *t* while  denotes protein *i* is not in predicted complex *k*. In this study, the value of *τ* is set to 0.3, the same as in [14] (In next section, we will discuss how changing this parameter can affect the final results). In addition, we only consider predicted complexes with at least three proteins [12]. The detailed TS-OCD algorithm of identifying temporal protein complexes is illustrated in Additional file 1: Figure S4.

#### Merging temporal protein complexes

Since the dynamic PPI networks, *G*^(*t*)^(1 ≤ *t* ≤ *T*), contain a considerable fraction of stable interactions, some complexes detected across different time points will be quite similar. Thus we needed to merge those similar complexes to generate a final set of predicted complexes. Note we will only match and merge those very similar complexes but still maintain those time-specific complexes that occur only at certain dynamic PPI networks.

In this paper, we use a Nonnegative Matrix Factorization (NMF) model to merge similar temporal protein complexes, which provides a low rank approximation of a nonnegative matrix and has been widely used as a clustering method [35, 36]. After we compute a series of protein-complex assignment matrices , a combined protein-complex assignment matrix *Y* is defined as *Y* = [*H*^(1)^^⋆^,…,*H*^(*T*)^^⋆^]. According to this definition, matrix *Y* = [*Y*_*il*_] ∈ {0,1}^*N*×*L*^ contains *N* rows and *L* = *r*_1_ + … + *r*_*T*_ columns, each of which represents a complex detected at the corresponding time point, where *Y*_*il*_ = 1 if protein *i* belongs to complex *l* and *Y*_*il*_ = 0 otherwise. Our objective is to detect similar complexes from *Y*.

**Figure 1:**
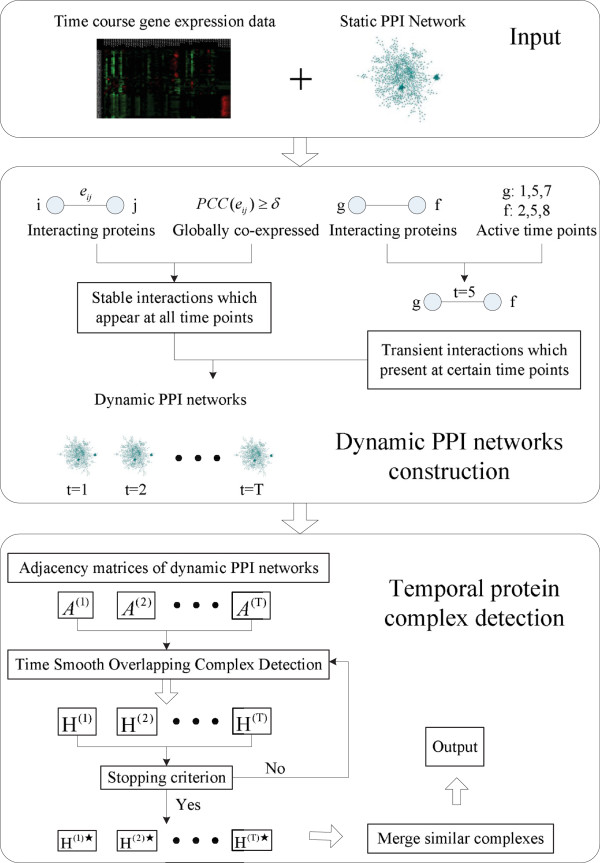
**Schematic overview of the algorithm.** TS-OCD consists of two stages. First, it constructs dynamic PPI networks by integrating physical protein interaction data and time-course gene expression data. Second, it detects temporal protein complexes from the constructed dynamic PPI networks.

Assume there are *K* final complexes inherent in *Y*, we formulate the nonnegative matrix factorization of *Y* as:
7

where ,  and  denotes the set of nonnegative real numbers. The model is solved by DTU:Toolbox [37] via multiplicative update method [34]. After calculating the solutions  and , we need to infer the group relationship of each complex *l* from . Here, complex *l* is assigned to a group *z* if . Finally, we merge complexes within same groups and obtain the final set of predicted complexes. The flow-chart of our proposed algorithm, including 2 key steps, namely, constructing dynamic PPI networks, and detecting temporal protein complexes, is shown in Figure 1.

## Results and discussion

In this section, we will first introduce the data, evaluation metrics and parameter settings. Then, we will present detailed experimental results.

### Data, evaluation metrics and parameter settings

#### Protein interaction networks and time course gene expression data

Two yeast PPI networks have been employed for evaluating the performance of various complex detection methods, including 1) **DIP** PPI network [38], and 2) **BioGrid** PPI network (version 3.1.77) [39]. DIP data contain 21592 interactions among 4850 proteins, while BioGrid contain 59748 interactions among 5640 proteins.

Note that both DIP and BioGrid are aggregates of protein interactions obtained under different conditions or time points. In order to extract dynamic PPI networks from these datasets, we have used yeast metabolic cycle (YMC) gene expression microarrays [40] to infer stable and transient interactions. YMC reports the expression values for 3552 significant periodic genes [40] at 12 time points (i.e. *T* = 12 in our experiments, there are about 25 minutes per each time interval) over three successive cycles. The raw data are available on Gene Expression Omnibus (GEO) [41] with the accession number GSE3431. Similar to [22], in our experiment, the average expression value of each gene at the same time point of three cycles is used as its expression value at that time point. Among the 3552 genes, 2389 occur in DIP and 3057 occur in BioGrid. Thus, we retain these genes and their corresponding interactions in DIP and BioGrid respectively.

#### Gold standard protein complexes

To measure whether the predicted complexes match with known experimentally determined protein complexes, we have chosen two benchmark complex sets as our gold standard. They are derived from CYC2008 [42] and MIPS [43] respectively. For both gold standard sets, to avoid selection bias, we filter out the proteins that are not involved in the two PPI networks. Moreover, we only consider complexes with at least 3 proteins.

#### Metrics

We utilize two independent quality criteria, namely PR metric [44] and *f*-measure [6], to evaluate the performance of various complex detection methods. Among these two measures, PR metric judge how well the predicted complexes match with known complexes mainly by considering the percentage of their overlapping proteins. *f*-measure is the harmonic mean of *recall* and *precision* where *recall* measures how many known gold standard complexes are matched by the predicted complexes, while *precision* measures how many predicted complexes are matched with known complexes. The two metrics have complementary strengths and they could thus evaluate the prediction performance from different perspectives. In addition, they all give a value in the range of 0-1, where the higher values indicate the better performance.

Please refer to the Additional file 1 for more detailed description about the two PPI networks, two gold standard complex sets, as well as two evaluation metrics.

#### Parameter setting

When extracting dynamic PPI networks from given static PPI networks, we distinguish stable interactions from transient interactions by calculating the PCCs of their associated gene pairs’ expression values across all time points (i.e., *PCC*(*e*_*ij*_)). Physical interactions with PCC values greater than a certain cutoff *δ* are defined as stable interactions. To determine the cutoff threshold, we use the PCC values of all the physical interactions and fit the PCC distribution with two parametric distributions, assuming one from the stable interactions and the other from the transient interactions.

**Figure 2:**
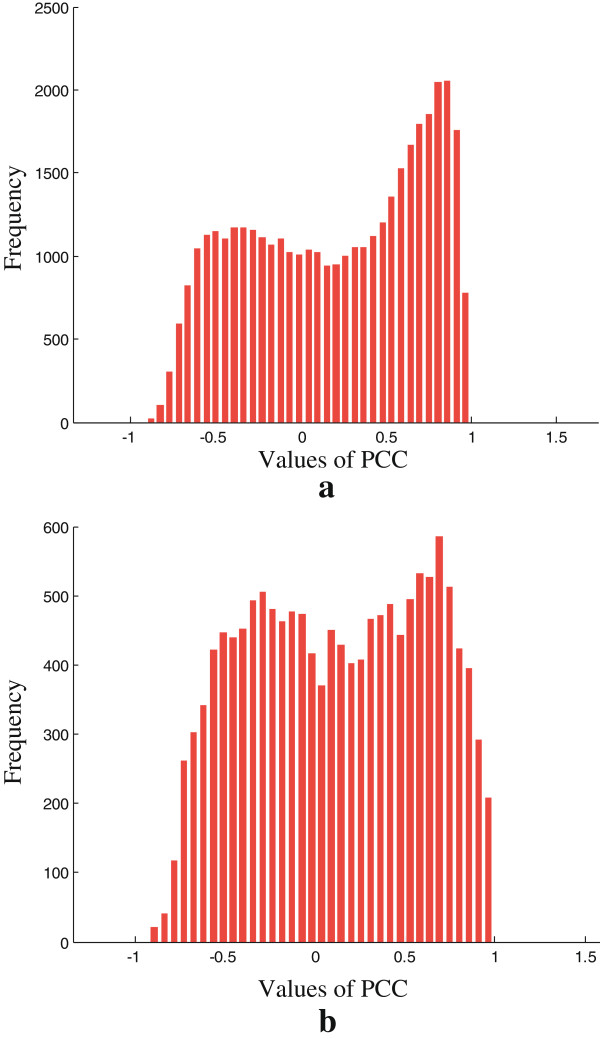
**The frequency distribution histogram of the PCC values.** The frequency distribution histogram of the PCC values of all interactions on **(a)** BioGrid and **(b)** DIP.

As shown in Figure 2(a), the frequency distribution histogram of the PCC values of all physical interactions in BioGrid shows that they can be sorted into two well separated classes (interactions in DIP have similar properties as shown in Figure 2(b)). Therefore, we assume that the data consist of two distributional components: a *η* proportion of Gaussian distributed stable interactions and a (1- *η*) proportion of another Gaussian distributed transient interactions, which is consistent with the observed data. The proposed Gaussian mixture model (GMM) has the following form:
8

where *η* is the proportion with values between 0 and 1,  is the Gaussian distribution with mean *μ*_1_ and variance .

**Figure 3:**
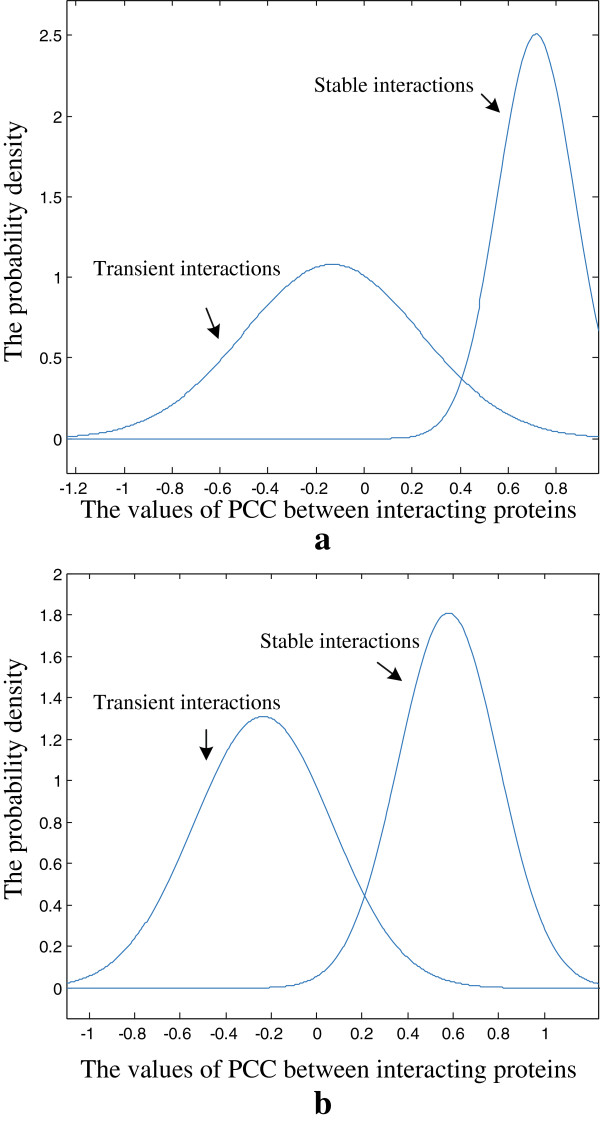
**The probability density functions learned from the GMMs.** The estimated probability density functions for the PCC values of transient interactions and stable interactions on **(a)** BioGrid and **(b)** DIP. For both BioGrid and DIP, the distribution on the left side corresponds to the estimated distribution for PCC values of transient interactions while the distribution on the right side corresponds to the estimated distribution for PCC values of stable interactions.

We use Expectation Maximization (EM) algorithm to estimate the parameters of the above two Gaussian distributions  for each dataset. The probability density functions learned from the BioGrid data and DIP data are shown in Figure 3(a) and (b) respectively. As shown in Figure 3, the two estimated distributions for each dataset are well separated. As stable interactions tend to be encoded by globally co-expressed gene pairs [27], the curve on the left side may correspond to the estimated distribution for PCC values of transient interactions while the curve on the right side may correspond to the estimated distribution for PCC values of stable interactions. From Figure 3, we can find that for both BioGrid and DIP, *δ* ∈ (0.2,0.4) can result in a relatively low rate of misclassification errors. Thus, we consistently keep *δ* = 0.3 in our experiments.

Both TS-OCD and NMF need to define the number of complexes, i.e. {*r*_1_,…,*r*_*T*_} and *K*. With our low rank constrain of each *U*^(*t*)^, we can give TS-OCD a relatively large values of *r*_*t*_ since the model could adaptively control the number of generated complexes. When merging similar temporal protein complexes via nonnegative matrix factorization, similar complexes likely to associate with same latent index and irrelevant latent indexes always obtain lower associations. As such, the value of *K* could also be relatively large since irrelevant dimensions will be filtered out. In this study, the values for *r*_*t*_ (*t* = 1,…,*T*), and *K* are set to 1000 since our algorithm is not sensitive to their values.

Recall that TS-OCD has three parameters *τ*, *λ* and *β* where *τ* is the threshold parameter, *λ* and *β* control the effects of the smooth regularization term *R* and low rank constrain respectively. To fully understand how these three parameters affect the performance of TS-OCD, we perform the sensitivity studies. Particularly, we first keep *τ* = 0.3 and run TS-OCD with different combination values of *λ*(*λ* ∈ {2^-7^,2^-6^,…,2^1^}) and *β*(*β* ∈ {2^0^,2^1^,…,2^6^}) and assess how well the predicted complexes match with gold standard sets. Then we fix the values of *λ* and *β* which result in the best performance, and study the effect of *τ* on the performance of TS-OCD by setting *τ* = 0.1,0.2,…,0.6, respectively. Moreover, in order to verify the generalization of TS-OCD, we select their best parameter values by testing the performance of TS-OCD on DIP and BioGrid in terms of *f*-measure with respect to the reference set MIPS. Therefore, the performance of TS-OCD on DIP and BioGrid with respect to the other reference set CYC2008 can well validate the general performance of TS-OCD.

**Figure 4:**
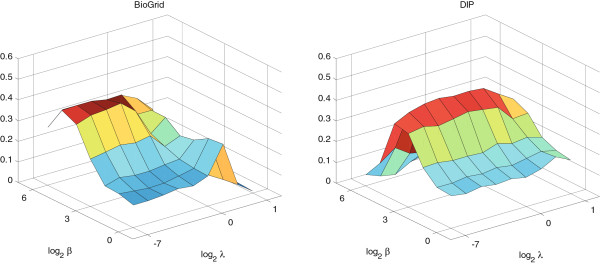
**The effect of** ***λ*** **and** ***β*** **.** Performance of TS-OCD on protein complex detection with different values of *λ* and *β* measured by *f*-measure with respect to MIPS on BioGrid and DIP. The *x*-axis denotes the value of log *λ*, the *y*-axis denotes the value of log *β*, and the *z*-axis denotes the value of *f*-measure.

**Figure 5:**
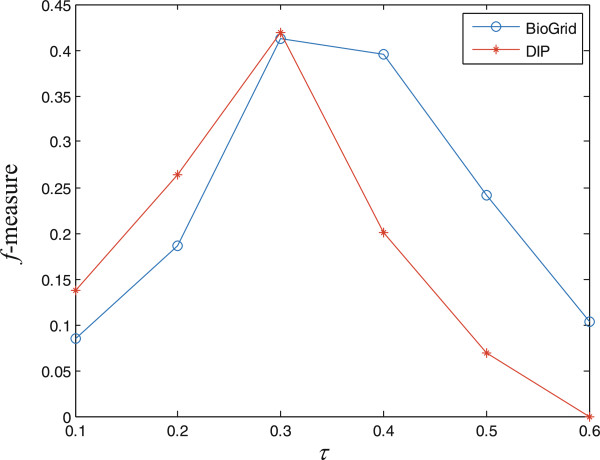
**The effect of** ***τ*** **.** Performance of TS-OCD on protein complex detection with different values of *τ* measured by *f*-measure with respect to MIPS on BioGrid and DIP. The *x*-axis denotes the value of *τ* and the *y*-axis denotes the value of *f*-measure.

From Figure 4 we observe that for a fixed value of *λ*, as the value of *β* increases, the *f*-measure increases initially and decreases after reaching the maximum. Similarly, for a fixed value of *β*, as the value of *λ* increases, *f*-measure increases initially and decreases after reaching the maximum. Thus both *β* and *λ* contribute to improve the performance of TS-OCD. Overall, we find that for DIP and BioGrid, *λ* ∈ [2^(-4)^,2^(-3)^] and *β* ∈ [2^4^,2^5^] result in competitive results. On the other hand, we can find from Figure 5 that TS-OCD is sensitive to *τ*. Overall, TS-OCD achieved best performance when *τ* = 0.3. In order to avoid evaluation bias and over-estimation of the performance, we do not tune the parameters for a particular dataset and fix *τ* = 0.3, *λ* = 2^(-4)^ and *β* = 2^4^ in the following experiments. Nevertheless, it is worthy to mention that better performance may be achieved if the parameters are tuned for a particular PPI dataset or for a particular complex reference set.

### Comparison with static complex detection methods

In order to demonstrate the benefits of using our constructed *dynamic* PPI networks, we compare our proposed TS-OCD method with five state-of-the-art algorithms, namely ClusterONE [12], MCL [8], MINE [45], COACH [46] and SPICi [47], which are originally designed for detecting protein complexes from *static* PPI networks. We apply these five algorithms on available static PPI networks (full PPI networks which are assembled by stable interactions and transient interactions) and apply TS-OCD on our constructed dynamic PPI networks respectively, and evaluate the predicted complexes in terms of two metrics with respect to two gold standards. Note optimal parameters are set for MCL, MINE, COACH and SPICi to generate their best results (in terms of *f*-measure with respect to MIPS and CYC2008) while ClusterONE has used the default parameters set by the authors. For detailed parameter settings of these five algorithms, please refer to Additional file 1.

**Figure 6:**
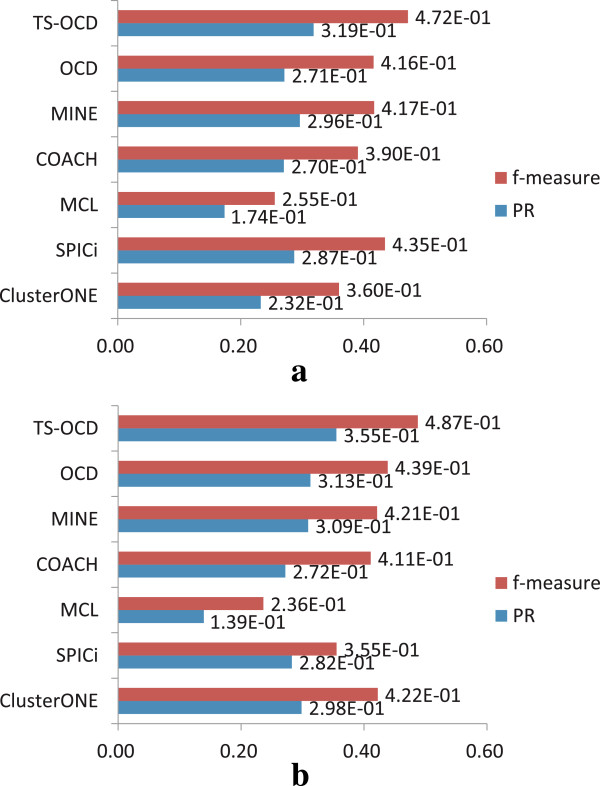
**Comparison with other static protein complex detection methods.** Comparison results on two PPI networks in terms of PR and f-measure with respect to CYC2008. **(a)** DIP data. **(b)** BioGrid data.

We also apply TS-OCD on static PPI networks, i.e., discard the smooth regularization term in the objective function (5) and take the static PPI networks as input (we denote it as OCD). For fair comparison, optimal parameters are also set for OCD to generate its best results. In addition, we discard their predicted complexes with less than three proteins, for all the 7 methods. Figure 6 shows the comparative performance of 7 different algorithms on two PPI networks with respect to benchmark complex set CYC2008. Moreover, Table 1 shows the size distribution of complexes detected by various algorithms, and the values of *recall* and *precision* for each algorithm.

**Table 1:** **Comparative results of various algorithms on two PPI networks using CYC2008 as benchmark**

Network	Algorithm	# complexes	avg size	std	CYC2008		
					***Precision***	***Recall***	***f***-measure
BioGrid	ClusterONE	260	5.97	4.93	0.312	0.655	0.422
	SPICi	136	6.06	5.23	0.294	0.448	0.355
	MCL	264	8.53	29	0.167	0.405	0.236
	COACH	182	7.94	7.48	0.324	0.560	0.411
	MINE	219	6.06	7.48	0.311	0.655	0.421
	OCD	209	6.62	6.19	0.332	0.647	0.439
	TS-OCD	606	7.42	6.95	0.363	0.741	0.487
DIP	ClusterONE	166	4.18	1.56	0.301	0.447	0.360
	SPICi	78	4.26	1.22	0.453	0.417	0.435
	MCL	338	5.21	5.04	0.163	0.592	0.255
	COACH	151	4.45	2.12	0.305	0.544	0.390
	MINE	121	4.03	1.91	0.355	0.505	0.417
	OCD	82	4.24	1.93	0.415	0.417	0.416
	TS-OCD	254	3.99	1.43	0.429	0.524	0.472

Here "# complexes" denotes the number of detected complexes, "avg size" and "std" denote the average size and standard deviation of the detected complexes.

As shown in Figure 6, for both DIP and BioGrid, our TS-OCD outperforms other 5 existing methods in terms of two metrics based on the benchmark CYC2008 (we have similar results with respect to MIPS benchmark in Additional file 1: Figure S2). For instance, on DIP data, TS-OCD achieves the highest *f*-measure 0.472, which is 8.5% higher than the second best f-measure 0.435, achieved by SPICi. On BioGrid data, TS-OCD also achieves the highest f-measure 0.487, which is 15.4% higher than the second best f-measure 0.422 achieved by ClusterONE. In Table 1, we can find that TS-OCD achieves a good performance due to its high *recall* and *precision*. Additional file 1: Table S1 in the Additional files shows similar results with respect to the MIPS benchmark. Interestingly, we also observe that OCD achieves better performance than the above 5 existing algorithms on both DIP and BioGrid data. Thus, even without using time-course gene expression information, our method could also be utilized to better detect complexes from static PPI networks. On the other hand, by taking into account the temporal gene expression data to construct dynamic PPI networks, our method is able to capture time-evolving protein complexes and thus detect complexes much more accurately.

### Comparison with dynamic complex detection methods

Recently, Park *et al.* [28] proposed Dynamical Hierarchical Agglomerative Clustering (DHAC) method to detect protein complexes from dynamic PPI networks, with two different versions, i.e. DHAC-const and DHAC-local. The existing methods, such as ClusterONE, SPICi, MCL, COACH and MINE, can also be adapted to handle each of the dynamic PPI networks across different time points. For fair comparison, we have also applied nonnegative matrix factorization (NMF) to merge those clusters predicted by each method into their own final predicted complex results.

**Figure 7:**
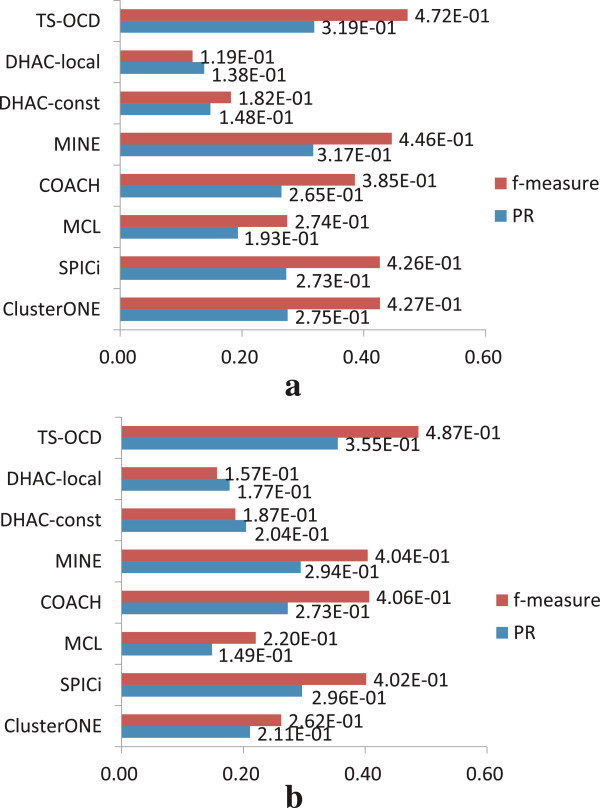
**Comparison with other temporal protein complex detection methods.** Comparison results on *dynamic* PPI networks with respect to CYC2008. **(a)** DIP data. **(b)** BioGrid data.

Figure 7 illustrates the comparison among all the above algorithms with respect to CYC2008 (the detailed comparative results of various algorithms are listed in Additional file 1: Table S2). We observe that TS-OCD achieves best performance than existing algorithms consistently in terms of the two measures across DIP and BioGrid data (similar results obtained with respect to MIPS benchmark in Additional file 1: Figure S3). Moreover, some existing algorithms combined with our NMF model obtain notable gains in prediction accuracy on the dynamic PPI networks. For example, ClusterONE achieves 0.360 *f*-measure on the *static* DIP data, but it increases to 0.427 on the *dynamic* DIP data. Similarly, SPICi achieves 0.355 *f*-measure on the *static* BioGrid data, but it increases to 0.402 on the *dynamic* BioGrid data. Therefore, the information contained in dynamic PPI networks are indeed useful and they complement to the static PPI data for better complex detection.

Besides NMF, there are also some other algorithms that could be used to merge those similar complexes. Another widely used method is based on the overlap between different complexes. To study the effectiveness of NMF in merging those similar complexes, we also apply the reduction strategy proposed by Wang *et al.* [22] to merge those similar complexes. Since their method is based on the overlap between different complexes, how to decide the value of the similarity threshold is an important problem. In this study, the similarity threshold is set to be 0.65 as recommended by the authors. For more details about the reduction strategy proposed by Wang *et al.*, please refer to [22]. The results of using the reduction strategy proposed by Wang *et al.* are shown in Additional file 1: Table S3. We can find from Additional file 1: Table S3 that TS-OCD can still achieve the best performance. Furthermore, we could find that NMF is more accurate in merging those similar complexes, since better precision and recall are obtained when using NMF as the reduction strategy.

### Detecting multi-functional proteins

Protein complexes predicted by various methods can be used for protein function prediction [48] – a unknown protein can be assigned with its involved complex’s functions. However, multi-functional proteins carry out different functions by interacting with different partners at different time points [11]. It is thus a challenging task for traditional complex detection methods to predict multi-functional proteins based on the static view of PPI networks, which cannot reflect the dynamic nature of real PPI networks. Our proposed TS-OCD method, on the other hand, can handle this task well, as it is specially designed to detect time-evolving overlapping protein complexes by integrating PPI data with temporal gene expression data. Next, we present an interesting case study to show how the complexes predicted by our method help to detect and analyze multi-functional proteins.

**Figure 8:**
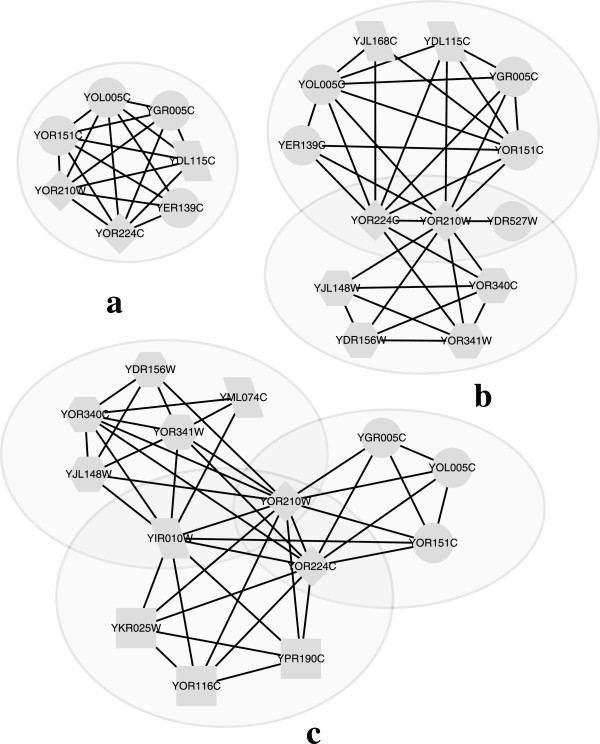
**The estimated probability density functions.** Interaction map of DNA-directed RNA polymerase I, II, III complexes detected by 3 different algorithms on BioGrid. Proteins are labeled according to the complexes they belong to: hexagon nodes represent RNA polymerase I, circle nodes represent RNA polymerase II, rectangle nodes represent RNA polymerase III, diamond nodes represent proteins shared by all the three complexes and parallelogram nodes represent proteins with other functions. Shaded areas represent the clusters detected by **(a)** SPICi, **(b)** ClusterONE, and **(c)** TS-OCD.

YOR210W is a multi-functional protein which is shared by three complexes, namely, the DNA-directed RNA polymerase I, DNA-directed RNA polymerase II, and DNA-directed RNA polymerase III [39, 42]. Employing SPICi [47] (designed for non-overlapping complex detection) and ClusterONE [12] (designed for overlapping complex detection) on the static BioGrid data, we can find that only one complex detected by SPICi includes YOR210W as shown in Figure 8(a), so that SPICi can only assign one function, i.e., DNA-directed RNA polymerase II to it. From Figure 8(b), ClusterONE is better than SPICi and it can assign the protein with two functions, namely DNA-directed RNA polymerase I and DNA-directed RNA polymerase II (for more examples, please refer to Additional file 1). Finally, our proposed TS-OCD detect all the above 3 overlapping complexes in Figure 8(c) and thus we are able to predict proteins’ multi-functions more accurately.

Moreover, when running SPICi on dynamic BioGrid PPI networks, it predicts two different complexes with YOR210W, i.e., {YOR210W, YOR224C, YGR005C, YOR341W, YOR340C, YDR156W, YJL148W} and {YJL164C, YER125W, YHL024W, YOR151C, YOL005C, YGR005C, YOR210W, YOR224C}. These two complexes match with both the RNA polymerase I and II complexes. Recall that SPICi can only generate one cluster based on the static BioGrid data involving YOR210W. Hence, dynamic networks indeed provide us with more insights into the proteins’ temporal activities for dynamic complex formation.In addition, as shown in Figure 8(c), TS-OCD predicts a novel protein YIR010W for both DNA-directed RNA polymerase I and III complexes. As protein YIR010W interacts with most members in RNA polymerase I, and all members in RNA polymerase III, we infer that YIR010W is likely to be multi-functional and highly related to RNA polymerase. By checking and browsing the literature, we find that YIR010W is a component of MIND kinetochore complex which is required for correct chromosome alignment and is related to the assembly of the RNA polymerase complex.

## Conclusion

In real biological environments, protein interaction networks are not static – they dynamically change across different time points [29]. Many existing protein complex mining methods, however, detect protein complexes from the overly simplified *static* PPI network model, which can not capture the inherent dynamic nature of protein interactions as well as modular temporal protein complexes.

Temporal protein complexes are typically constructed by the dynamic assembly or disassembly of proteins to perform various biological functions [49]. As they can better reflect the real-world dynamic molecular mechanisms inside the cellular systems, it is thus crucial to detect them by systematically analyzing dynamic PPI networks. Although a few methods have been proposed to identify temporal protein complexes by applying static complex detection methods for each individual time point, they fully ignore the correlations between the consecutive dynamic protein networks and thus cannot work well. In addition, these methods can not generate overlapping protein complexes and they do not reflect the biological observation that proteins frequently involve in multiple protein complexes [6] to play diverse biological functions.

To address these problems, in this study, we introduce a novel Time Smooth Overlapping Complex Detection model (TS-OCD) to detect overlapping temporal protein complexes from the dynamic PPI networks. Particularly, we construct a series of dynamic PPI networks by detecting stable interactions and transient interactions via integrating protein interaction data and gene expression data. Our proposed TS-OCD allows individual complex to be assembled and disassembled across different time points. Furthermore, with the smoothness regularization term, our model can detect conserved protein complexes that play fundamental roles in cellular systems. The analysis on real biological data shows that our proposed TS-OCD significantly outperforms existing state-of-the-art temporal complex detection methods. Furthermore, with the constructed dynamic PPI networks, our method could detect multi-functional proteins more correctly. All the experimental results, including the predicted stable complexes and temporal complexes, are shown in Additional files 2 and 3. We also investigate the benefits of using the smoothness regularization term by comparing the performance of our model without the smoothness regularization term. Our experimental results show that with the smoothness constrain, our method could detect temporal protein complexes more accurately, as we can better consider the conserved protein interactions between the consecutive networks. The detailed comparison are shown in Additional file 1.

In summary, compared with existing methods, our model has the following advantages:

-  We have distinguished two different types of protein interactions for constructing dynamic PPI networks. In particular, the stable interactions are reserved across different time points to serve as the backbone of the protein interaction networks, while transient interactions are only presented under certain conditions and thus occurred in dynamic part of PPI networks.
-  It allows the dynamic assembly process, i.e. individual complex to be assembled and disassembled across different time points. In addition, with smoothness regularization, it prevents the value of the assigned co-complex similarity for proteins with stable interactions from changing too dramatically.
-  It generates the overlapping temporal protein complexes, which clearly reflect the biological reality on proteins’ multi-functional roles.
-  Finally, our proposed method is unsupervised and thus is generic enough to apply for the dynamic complex detection of other species.

The computational complexity for updating *H*^(*t*)^ is *O* (*N*^2^*r*_*t*_), where *N* is the number of proteins, and *r*_*t*_ is the number of complexes at time *t*. Thus the overall time cost of TS-OCD is *O* (*N*^2^(*r*_1_ + … + *r*_*T*_)*I*), where *T* is the number of time points and *I* is the number of iterations. In practice the time cost will be much smaller since *H*^(*t*)^ is sparse and the number of proteins at each time point is less than *N*.

Applying our proposed TS-OCD method on dynamic PPI networks could effectively track the underlying dynamic modular organization and provide a new biological knowledge and insights about the molecular systems. In this study, we use time-course gene expression data to help construct dynamic PPI networks since it is one of the most abundant data that include the temporal information of proteins in the gene level. However, as it contains noisy information, the performance of our proposed algorithm could be limited by its poor quality. Moreover, there are a few of other related information sources, including a collection of genomics, functional genomics, genetics studies and their corresponding result datasets, biological pathway databases, cellar compartment information and biomedical ontologies. As such, in our future work, we will study how to reduce the noise in the gene expression data as well as to incorporate other biological evidences for constructing more accurate dynamic PPI networks that could lead to further performance improvements for detecting temporal protein complexes.

## Electronic supplementary material

Additional file 1: **Supplementary figures and text.** This section provides the supplementary figures referred in the main text and some text which describes the detailed inference of the solution to Time Smooth Overlapping Complex Detection model, the data sets and the evaluation methods we have used, the effects of different parts of the model, random start effect, convergence analysis, brief description and detailed parameter settings of the compared clustering algorithms. (PDF 1 MB)

Additional file 2: **Table S1.** Complete lists of the predicted protein complexes. (XLSX 116 KB)

Additional file 3: **Table S2.** Complete lists of the predicted stable complexes and temporal complexes. (XLSX 46 KB)
