# Supplementary material for: Detecting temporal protein complexes from dynamic protein-protein interaction networks
Source: BMC Bioinformatics. 2014 Oct 4;15(1):335. doi: 10.1186/1471-2105-15-335 (PMC4288635; doi:10.1186/1471-2105-15-335)
Supplement: Supplementary file 1 — Additional file 1: Supplementary figures and text. This section provides the supplementary figures referred in the main text and some text which describes the detailed inference of the solution to Time Smooth Overlapping Complex Detection model, the data sets and the evaluation methods we have used, the effects of different parts of the model, random start effect, convergence analysis, brief description and detailed parameter settings of the compared clustering algorithms. (PDF 1 MB) [file 12859_2014_6646_MOESM1_ESM.pdf]

Detecting temporal protein complexes from dynamic protein-protein interaction networks

Le Ou-Yang, Dao-Qing Dai, Xiao-Li Li, Min Wu, Xiao-Fei Zhang and Peng Yang

1 Supplementary Table

Table S1: Comparative results of various algorithms on two PPI networks using MIPS as benchmark.

Network	Algorithm	# complexes	avg size	std	MIPS		
					precision	*recall*	f-measure
BioGrid	ClusterONE	260	5.97	4.93	0.208	0.445	0.283
	SPICi	136	6.06	5.23	0.228	0.315	0.265
	MCL	264	8.53	29	0.133	0.301	0.184
	COACH	182	7.94	7.48	0.247	0.466	0.323
	MINE	219	6.06	7.48	0.201	0.473	0.282
	OCD	209	6.62	6.19	0.270	0.486	0.347
	TS-OCD	606	7.42	6.95	0.312	0.575	0.404
DIP	ClusterONE	166	4.18	1.56	0.247	0.331	0.283
	SPICi	78	4.26	1.22	0.387	0.331	0.357
	MCL	338	5.21	5.04	0.121	0.390	0.185
	COACH	151	4.45	2.12	0.245	0.426	0.311
	MINE	121	4.03	1.91	0.355	0.463	0.402
	OCD	82	4.24	1.93	0.390	0.331	0.358
	TS-OCD	254	3.99	1.43	0.397	0.449	0.421

Here "# complexes" denotes the number of detected complexes, "avg size" and "std" denote the average size and standard deviation of the detected complexes.

Table S2: Comparative results of various algorithms on two dynamic PPI networks.

Network	Algorithm	# complexes	avg size	std	CYC2008			MIPS		
					precision	*recall*	f-measure	*precision*	*recall*	f-measure
BioGrid	ClusterONE	844	8.66	8.07	0.160	0.724	0.262	0.114	0.479	0.184
	SPICi	572	7	6.65	0.285	0.681	0.402	0.255	0.610	0.360
	MCL	697	23.2	60.9	0.134	0.612	0.220	0.0962	0.466	0.159
	COACH	648	13.2	10.6	0.284	0.716	0.406	0.221	0.562	0.317
	MINE	550	8.55	12.2	0.278	0.741	0.404	0.234	0.541	0.327
	DHAC-const	109	32.3	25.3	0.413	0.121	0.187	0.413	0.116	0.182
	DHAC-local	130	31.9	22.7	0.323	0.103	0.157	0.362	0.116	0.176
	TS-OCD	606	7.42	6.95	0.363	0.741	0.487	0.312	0.575	0.404
DIP	ClusterONE	562	4.38	1.59	0.327	0.612	0.427	0.299	0.574	0.393
	SPICi	348	3.37	0.85	0.369	0.505	0.426	0.342	0.434	0.383
	MCL	763	5.76	5.23	0.170	0.718	0.274	0.136	0.544	0.218
	COACH	616	4.43	2.1	0.295	0.553	0.385	0.269	0.500	0.350
	MINE	399	5.21	3.97	0.358	0.592	0.446	0.318	0.566	0.407
	DHAC-const	240	22.5	19.4	0.179	0.184	0.182	0.225	0.154	0.183
	DHAC-local	113	29.5	26.7	0.186	0.0874	0.119	0.274	0.110	0.157
	TS-OCD	254	3.99	1.43	0.429	0.524	0.472	0.397	0.449	0.421

Here "# complexes" denotes the number of detected complexes, "avg size" and "std" denote the average size and standard deviation of the detected complexes.

Table S3: Comparative results of various algorithms on two dynamic PPI networks using the reduction strategy proposed by Wang *et al.*

Network	Algorithm	CYC2008				MIPS			
		PR	*precision*	*recall*	f-measure	PR	*precision*	*recall*	f-measure
BioGrid	ClusterONE	0.180	0.149	0.819	0.252	0.149	0.105	0.630	0.180
	SPICi	0.247	0.224	0.629	0.330	0.205	0.206	0.568	0.302
	MCL	0.129	0.141	0.655	0.232	0.113	0.097	0.466	0.161
	COACH	0.239	0.223	0.724	0.341	0.191	0.170	0.568	0.261
	MINE	0.245	0.220	0.759	0.341	0.206	0.182	0.589	0.278
	DHAC-const	0.153	0.232	0.112	0.151	0.147	0.232	0.110	0.149
	DHAC-local	0.137	0.197	0.103	0.136	0.135	0.237	0.116	0.156
	TS-OCD	0.278	0.288	0.724	0.412	0.211	0.247	0.555	0.342
DIP	ClusterONE	0.210	0.226	0.612	0.330	0.173	0.192	0.529	0.282
	SPICi	0.225	0.298	0.505	0.375	0.179	0.263	0.434	0.327
	MCL	0.158	0.141	0.699	0.235	0.129	0.116	0.537	0.191
	COACH	0.224	0.228	0.544	0.321	0.195	0.206	0.485	0.289
	MINE	0.255	0.273	0.563	0.367	0.209	0.265	0.559	0.359
	DHAC-const	0.112	0.099	0.175	0.127	0.108	0.110	0.140	0.123
	DHAC-local	0.114	0.107	0.068	0.083	0.121	0.173	0.088	0.117
	TS-OCD	0.271	0.406	0.476	0.438	0.225	0.366	0.434	0.397

2 Supplementary Figure

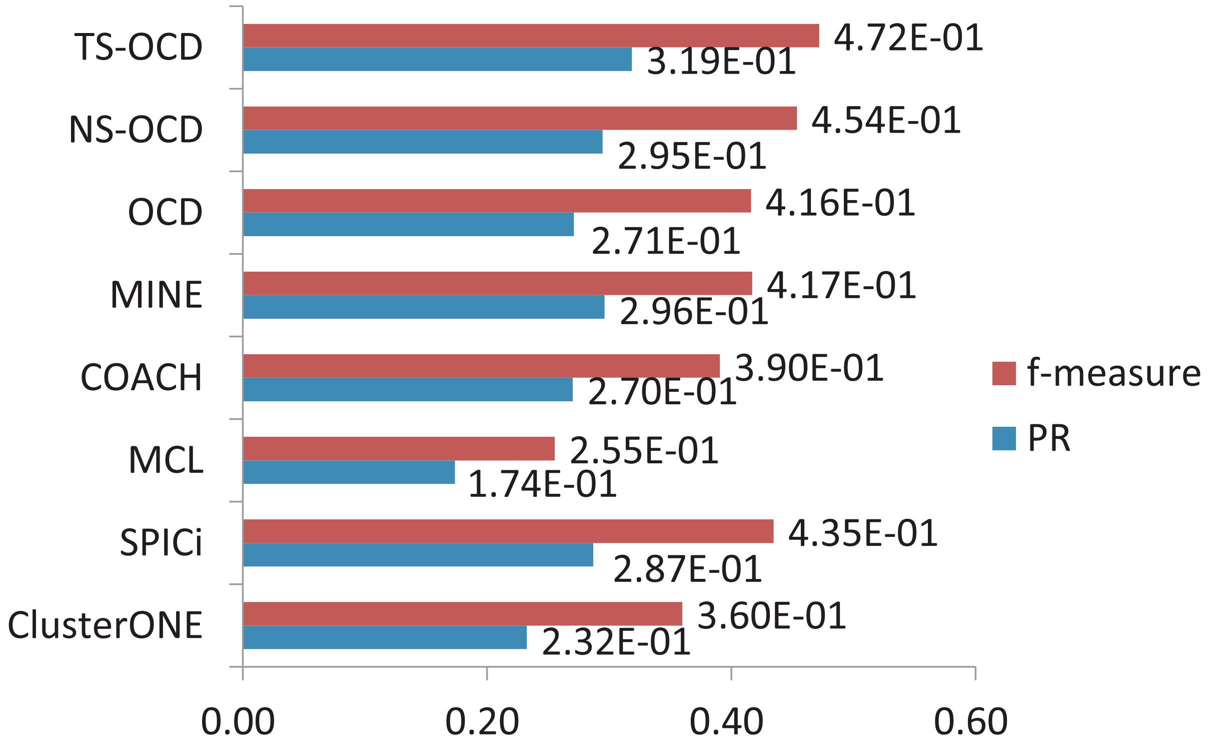

(a)

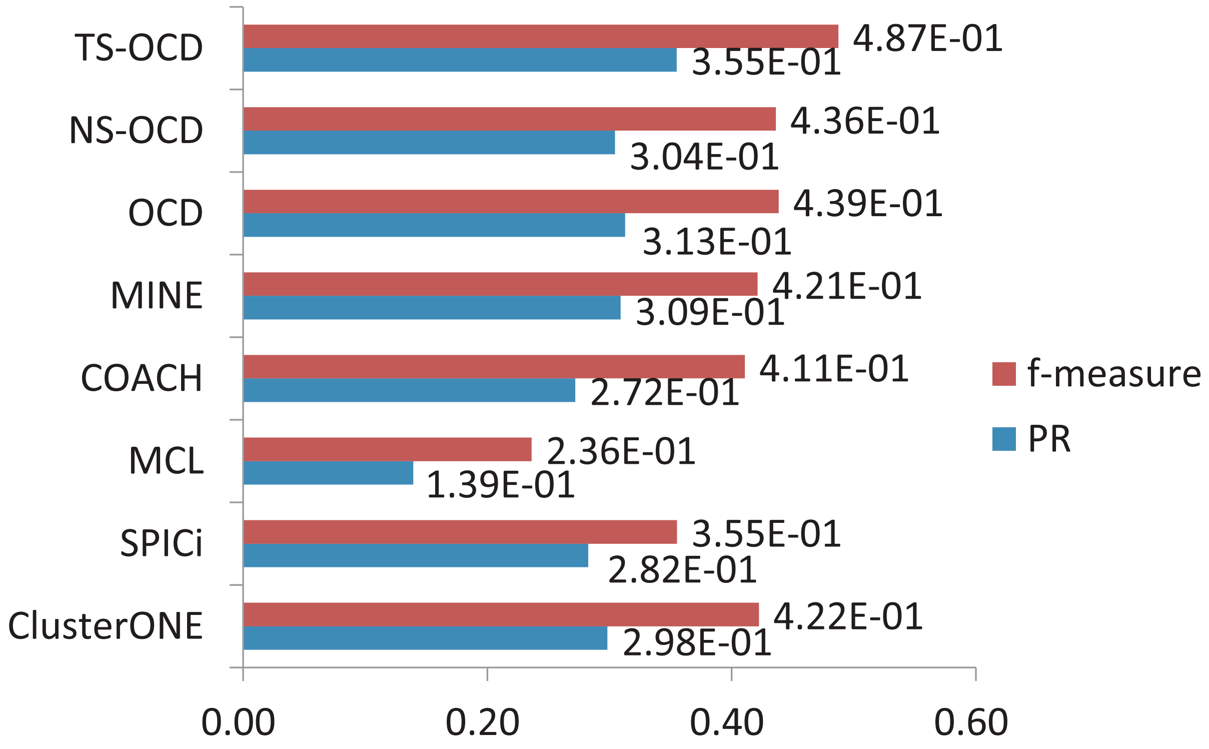

(b)

Figure S1: Performance of TS-OCD in comparison with ClusterONE, SPICi, MCL, COACH, MINE, OCD and NS-OCD on two PPI networks in terms of PR and f-measure with respect to CYC2008. (a) DIP. (b) BioGrid.

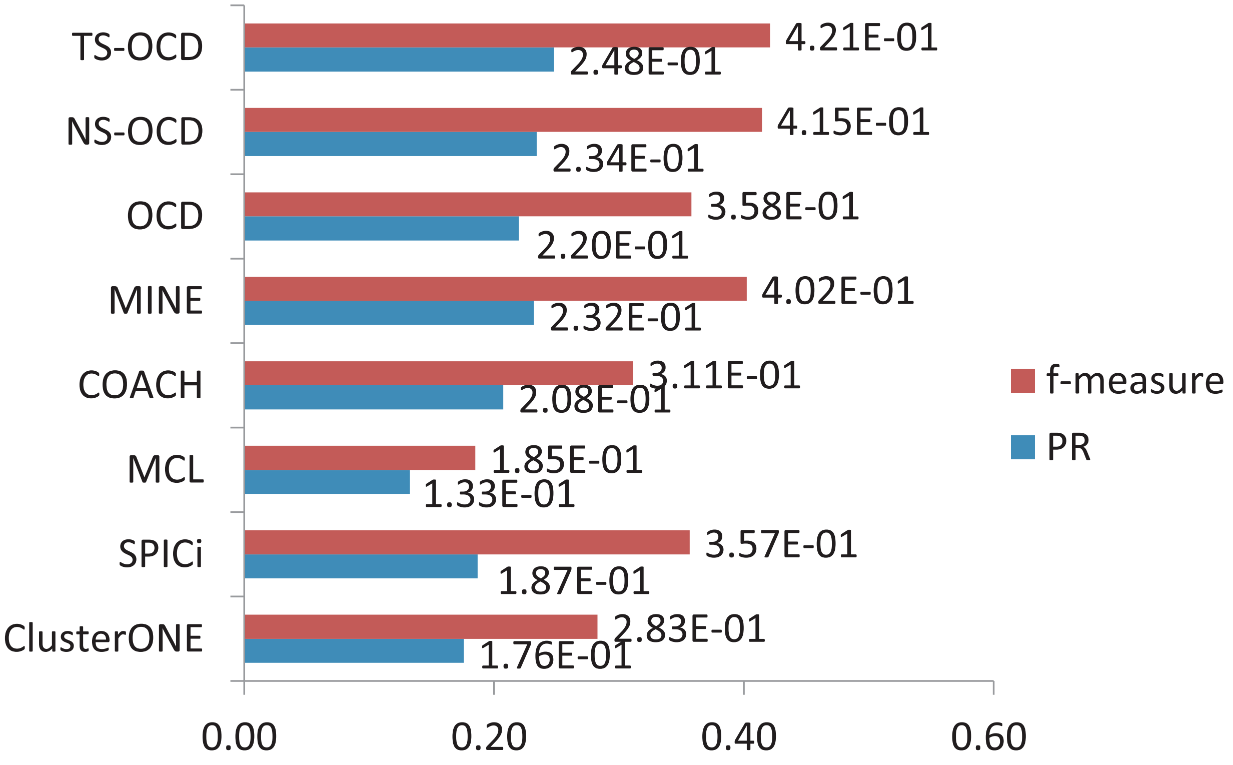

(a)

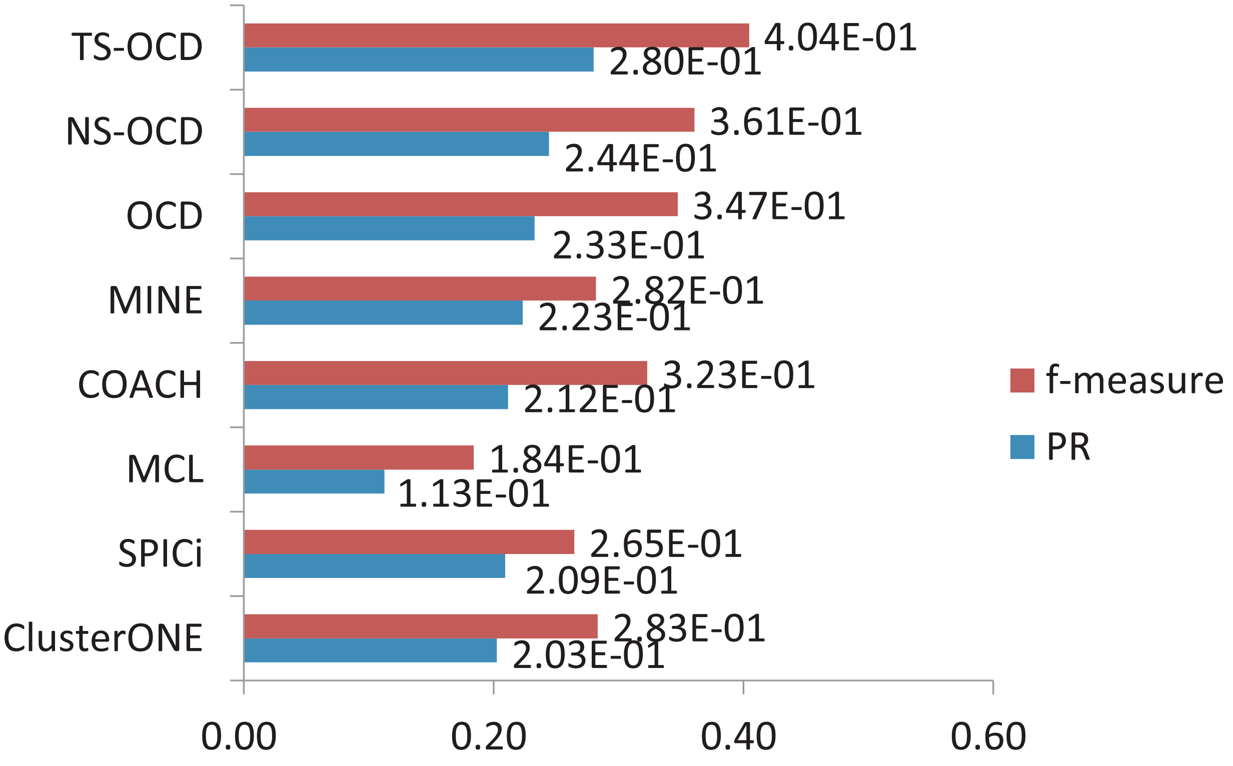

(b)

Figure S2: Performance of TS-OCD in comparison with ClusterONE, SPICi, MCL, COACH, MINE, OCD and NS-OCD in terms of PR and f-measure with respect to MIPS on (a) DIP and (b) BioGrid.

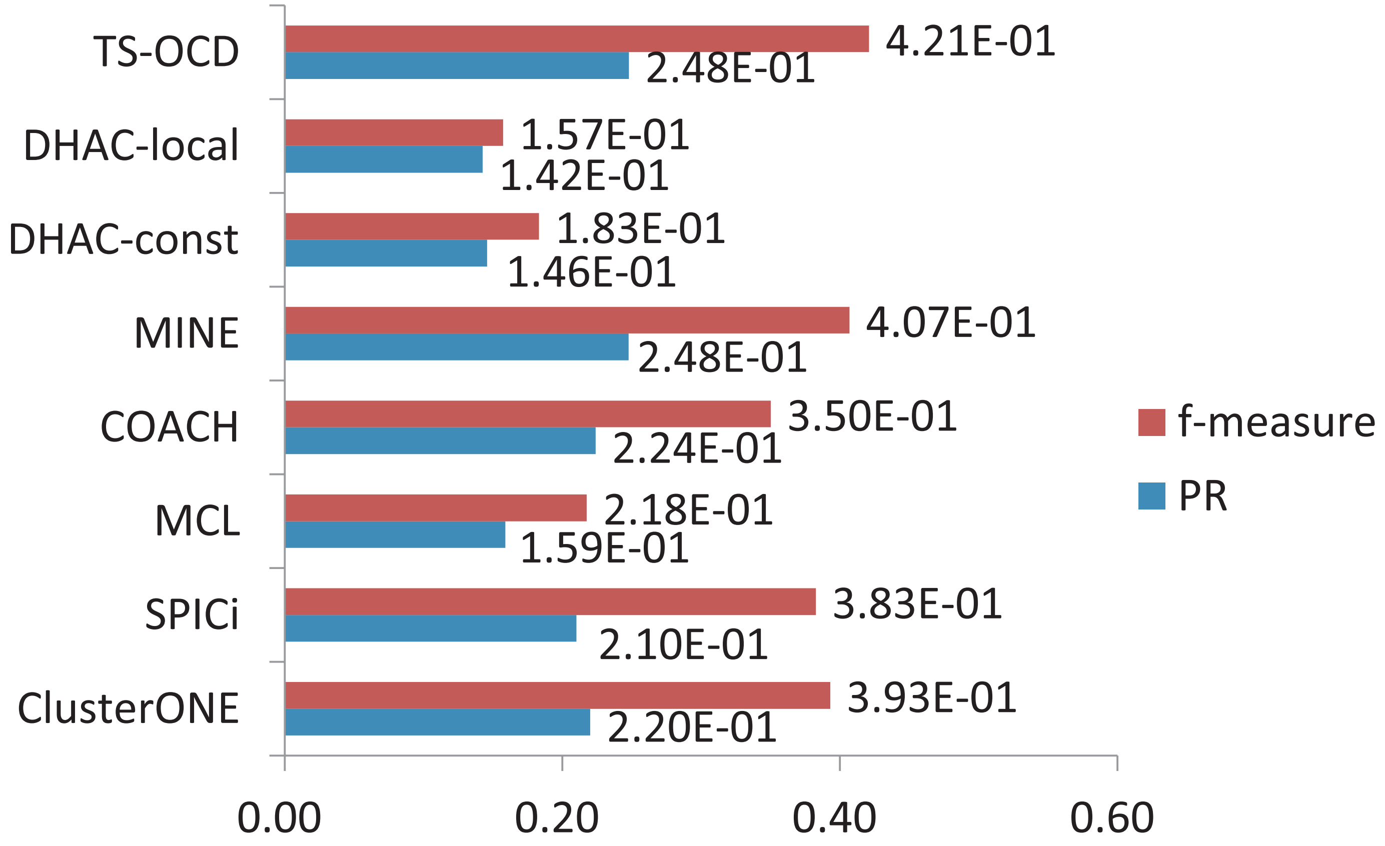

(a)

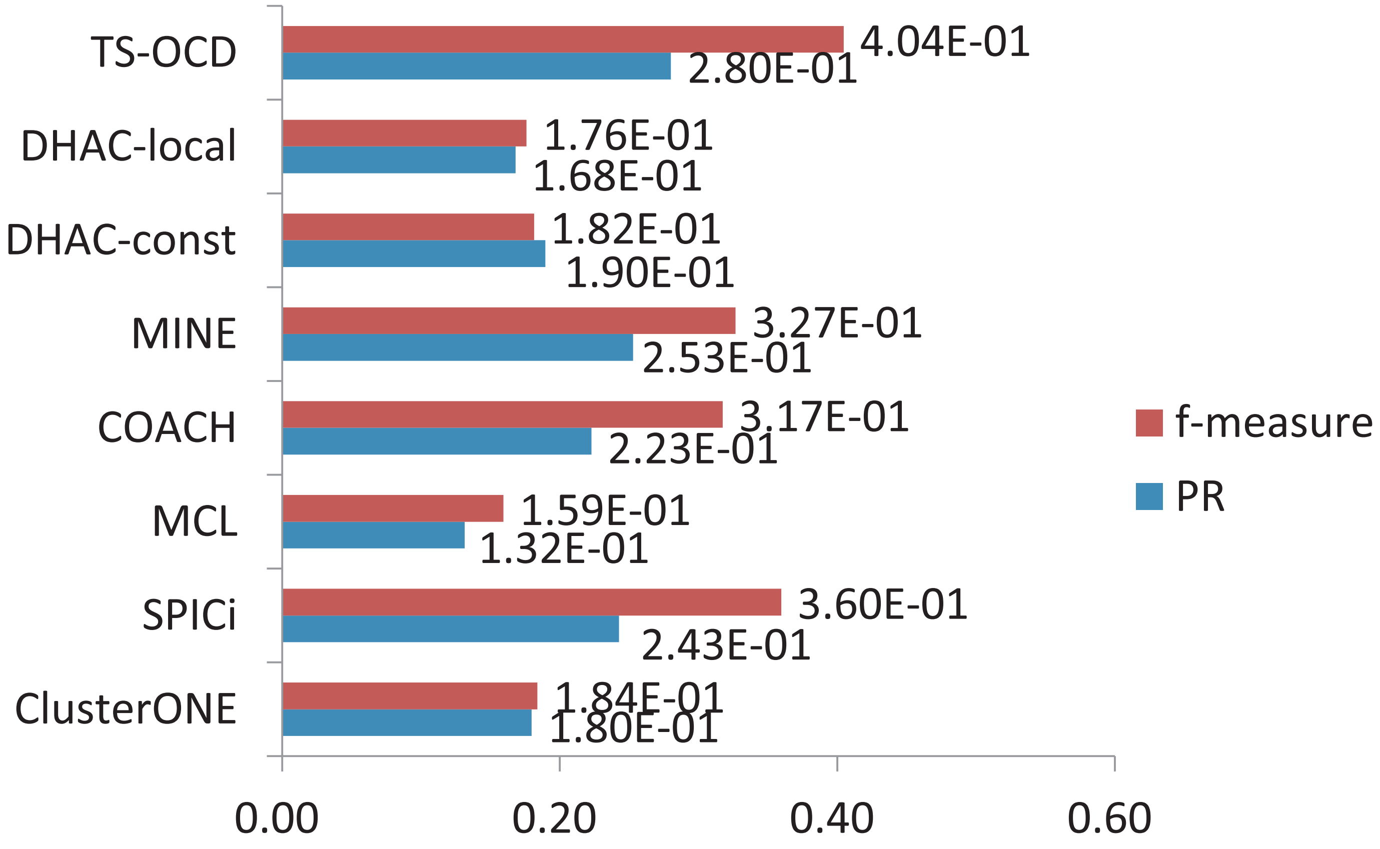

(b)

Figure S3: Comparison of the performance of TS-OCD, DHAC-const, DHAC-local, MINE, COACH, MCL, SPICi and ClusterONE in terms of PR and f-measure with respect to MIPS on dynamic PPI networks. (a) DIP. (b) BioGrid.

- **Input**

  $A^{(t)}$: Adjacency matrices of dynamic PPI networks;

  S: Matrix which represents stable interactions;

  r_t: Maximum number of possible complexes at time t;

  λ: Coefficient of smooth regularization;

  β: Coefficient of low rank regularization;

  τ: Threshold parameter for obtaining protein complex candidates.

- **Output**

  $H^{(t)\star}$: Protein-complex membership matrix at time t;

  s : Value of the objective function (1).

- **Main algorithm**

  1. Initialize matrices $H^{(t)}$ randomly;
  2. Update $H^{(t)}$ according to Equation (12), (13) and (14);
  3. Repeat Steps 2 until the relative change of $H^{(1)}$ and $H^{(T)}$ is less than 10^{-6} or times of iteration reach 200;
  4. Calculate the value s of the objective function (1);
  5. Obtain the protein-complex membership indication matrix $H^{(t)\star}$ according to Equation (6) in the main text;
  6. Filter out detected complexes which contain less than three proteins;
  7. Return $H^{(t)\star}$ and s.

Figure S4: The algorithm of detecting temporal protein complexes via TS-OCD.

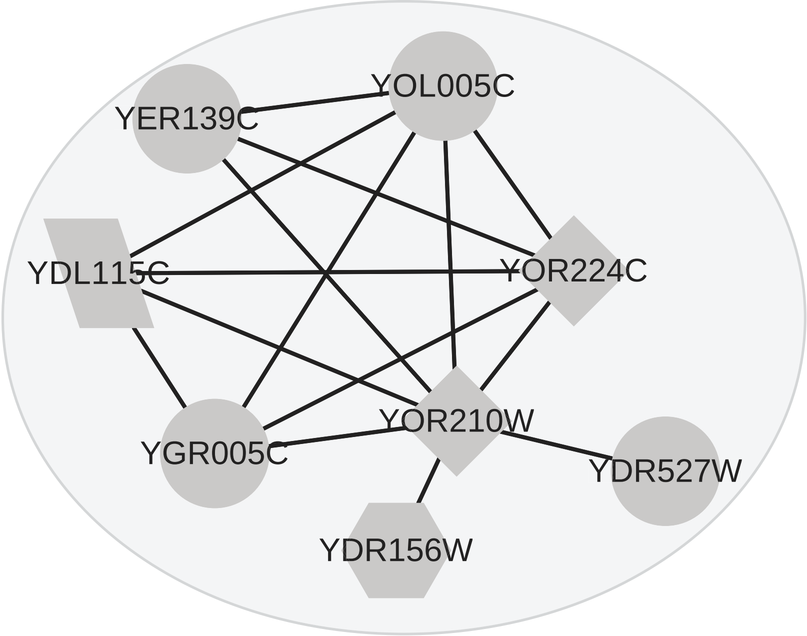

Figure S5: Interaction map of DNA-directed RNA polymerase I, II, III complexes detected by MCL on BioGrid. Proteins are labeled according to the complexes they belong to: hexagon nodes represent RNA polymerase I, circle nodes represent RNA polymerase II, rectangle nodes represent RNA polymerase III, diamond nodes represent proteins shared by all the three complexes and parallelogram nodes represent proteins with other functions. Shaded areas represent the clusters detected by MCL.

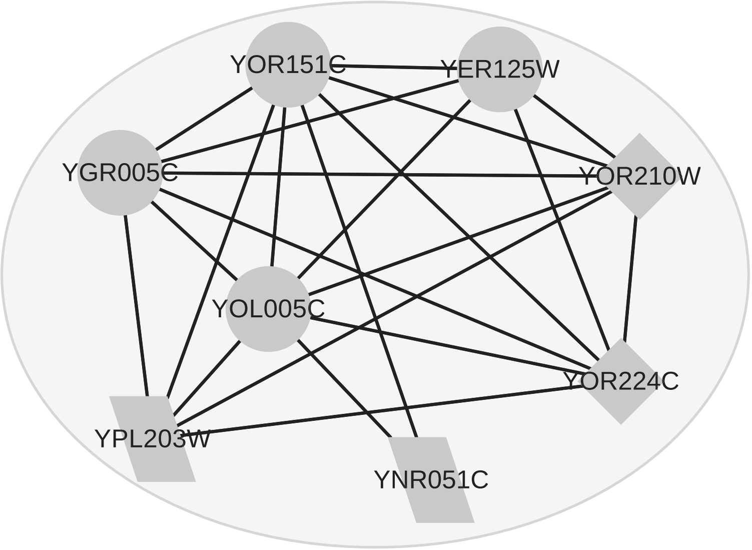

Figure S6: Interaction map of DNA-directed RNA polymerase I, II, III complexes detected by COACH on BioGrid. Proteins are labeled according to the complexes they belong to: hexagon nodes represent RNA polymerase I, circle nodes represent RNA polymerase II, rectangle nodes represent RNA polymerase III, diamond nodes represent proteins shared by all the three complexes and parallelogram nodes represent proteins with other functions. Shaded areas represent the clusters detected by COACH.

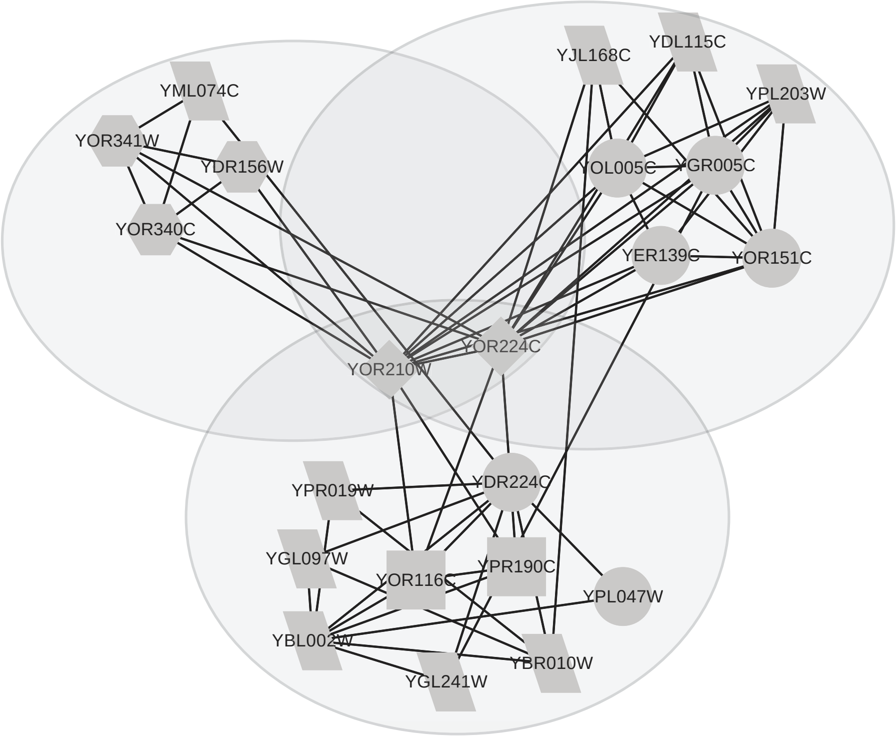

Figure S7: Interaction map of DNA-directed RNA polymerase I, II, III complexes detected by MINE on BioGrid. Proteins are labeled according to the complexes they belong to: hexagon nodes represent RNA polymerase I, circle nodes represent RNA polymerase II, rectangle nodes represent RNA polymerase III, diamond nodes represent proteins shared by all the three complexes and parallelogram nodes represent proteins with other functions. Shaded areas represent the clusters detected by MINE.

3 Supplementary Text

3.1 Model parameter estimation for TS-OCD

The objective function of TS-OCD is as follows:

$$\begin{cases} \min\limits_{\{H^{(t)}\}} & -\sum\limits_{t=1}^{T}\sum\limits_{i,j}\left(A_{ij}^{(t)}\log(\sum\limits_{k=1}^{r_t}H_{ik}^{(t)}H_{jk}^{(t)})-\sum\limits_{k=1}^{r_t}H_{ik}^{(t)}H_{jk}^{(t)}\right) \\ & +\lambda\sum\limits_{t=1}^{T-1}\sum\limits_{i,j}S_{ij}(\sum\limits_{k=1}^{r_{t+1}}H_{ik}^{(t+1)}H_{jk}^{(t+1)}-\sum\limits_{k=1}^{r_t}H_{ik}^{(t)}H_{jk}^{(t)})^2+\beta\sum\limits_{t=1}^{T}\|H^{(t)}\|_F^2. \\ s.t. & H^{(t)}\geq 0, t=1,\ldots,T, \end{cases} \tag{1}$$

where $\lambda \geq 0$ and $\beta \geq 0$ are the tradeoff parameters which control the balance between loss function and the regularization terms.

We utilize the multiplicative updating rule [6] to solve this nonnegative constrained optimization problem. Let $\Phi^{(t)} = [\phi_{ik}^{(t)}]$ be the Lagrange multipliers for constraint $H^{(t)} \geq 0, t = 1, \ldots, T$. Therefore, the Lagrange function $\mathcal{L}$ is as follows:

$$\begin{aligned} \mathcal{L}(H,\Phi) &= -\sum_{t=1}^{T}\sum_{i,j}\left(A_{ij}^{(t)}\log(\sum_{k=1}^{r_t}H_{ik}^{(t)}H_{jk}^{(t)})-\sum_{k=1}^{r_t}H_{ik}^{(t)}H_{jk}^{(t)}\right)+\beta\sum_{t=1}^{T}\|H^{(t)}\|_F^2+\sum_{t=1}^{T}\sum_{i=1}^{N}\sum_{k=1}^{K}\phi_{ik}^{(t)}H_{ik}^{(t)} \\ &\quad +\lambda\sum_{t=1}^{T-1}\sum_{i,j}S_{ij}(\sum_{k=1}^{r_{t+1}}H_{ik}^{(t+1)}H_{jk}^{(t+1)}-\sum_{k=1}^{r_t}H_{ik}^{(t)}H_{jk}^{(t)})^2. \end{aligned} \tag{2}$$

Taking the gradients of Lagrange function $\mathcal{L}$ with respect to $H_{ik}^{(t)}$, we could obtain:

$$\begin{aligned} \nabla_{H_{ik}^{(1)}}\mathcal{L} &= -2\sum_{j=1}^{N}A_{ij}^{(1)}\frac{H_{jk}^{(1)}}{\sum\limits_{k=1}^{r_1}H_{ik}^{(1)}H_{jk}^{(1)}}+2\sum_{j=1}^{N}H_{jk}^{(1)}+4\lambda\sum_{j=1}^{N}S_{ij}(\sum_{k=1}^{r_1}H_{ik}^{(1)}H_{jk}^{(1)})H_{jk}^{(1)} \\ &\quad -4\lambda\sum_{j=1}^{N}S_{ij}(\sum_{k=1}^{r_2}H_{ik}^{(2)}H_{jk}^{(2)})H_{jk}^{(1)}+2\beta H_{ik}^{(1)}+\phi_{ik}^{(1)}. \end{aligned} \tag{3}$$

and for $t = 2, \ldots, T-1$, we have:

$$\begin{aligned} \nabla_{H_{ik}^{(t)}}\mathcal{L} &= -2\sum_{j=1}^{N}A_{ij}^{(t)}\frac{H_{jk}^{(t)}}{\sum\limits_{k=1}^{r_t}H_{ik}^{(t)}H_{jk}^{(t)}}+2\sum_{j=1}^{N}H_{jk}^{(t)}+8\lambda\sum_{j=1}^{N}S_{ij}(\sum_{k=1}^{r_t}H_{ik}^{(t)}H_{jk}^{(t)})H_{jk}^{(t)} \\ &\quad -4\lambda\sum_{j=1}^{N}S_{ij}(\sum_{k=1}^{r_{t+1}}H_{ik}^{(t+1)}H_{jk}^{(t+1)}+\sum_{k=1}^{r_{t-1}}H_{ik}^{(t-1)}H_{jk}^{(t-1)})H_{jk}^{(t)}+2\beta H_{ik}^{(t)}+\phi_{ik}^{(t)}. \end{aligned} \tag{4}$$

and for $t = T$, we have:

$$\begin{aligned} \nabla_{H_{ik}^{(T)}}\mathcal{L} &= -2\sum_{j=1}^{N}A_{ij}^{(T)}\frac{H_{jk}^{(T)}}{\sum\limits_{k=1}^{r_T}H_{ik}^{(T)}H_{jk}^{(T)}}+2\sum_{j=1}^{N}H_{jk}^{(T)}+4\lambda\sum_{j=1}^{N}S_{ij}(\sum_{k=1}^{r_T}H_{ik}^{(T)}H_{jk}^{(T)})H_{jk}^{(T)} \\ &\quad -4\lambda\sum_{j=1}^{N}S_{ij}(\sum_{k=1}^{r_{T-1}}H_{ik}^{(T-1)}H_{jk}^{(T-1)})H_{jk}^{(T)}+2\beta H_{ik}^{(T)}+\phi_{ik}^{(T)}. \end{aligned} \tag{5}$$

Since the estimators of $H_{ik}^{(t)}$ need to satisfy $\nabla_{H_{ik}^{(t)}}\mathcal{L} = 0$, we can get:

$$\begin{aligned} \phi_{ik}^{(1)} &= 2\sum_{j=1}^{N}A_{ij}^{(1)}\frac{H_{jk}^{(1)}}{\sum\limits_{k=1}^{r_1}H_{ik}^{(1)}H_{jk}^{(1)}}-2\sum_{j=1}^{N}H_{jk}^{(1)}-4\lambda\sum_{j=1}^{N}S_{ij}(\sum_{k=1}^{r_1}H_{ik}^{(1)}H_{jk}^{(1)})H_{jk}^{(1)} \\ &\quad +4\lambda\sum_{j=1}^{N}S_{ij}(\sum_{k=1}^{r_2}H_{ik}^{(2)}H_{jk}^{(2)})H_{jk}^{(1)}-2\beta H_{ik}^{(1)}, \end{aligned} \tag{6}$$

and for $t = 2, \ldots, T-1$, we have:

$$\begin{aligned} \phi_{ik}^{(t)} &= 2\sum_{j=1}^{N}A_{ij}^{(t)}\frac{H_{jk}^{(t)}}{\sum\limits_{k=1}^{r_t}H_{ik}^{(t)}H_{jk}^{(t)}}-2\sum_{j=1}^{N}H_{jk}^{(t)}-8\lambda\sum_{j=1}^{N}S_{ij}(\sum_{k=1}^{r_t}H_{ik}^{(t)}H_{jk}^{(t)})H_{jk}^{(t)} \\ &\quad +4\lambda\sum_{j=1}^{N}S_{ij}(\sum_{k=1}^{r_{t+1}}H_{ik}^{(t+1)}H_{jk}^{(t+1)}+\sum_{k=1}^{r_{t-1}}H_{ik}^{(t-1)}H_{jk}^{(t-1)})H_{jk}^{(t)}-2\beta H_{ik}^{(t)}, \end{aligned} \tag{7}$$

and for $t = T$, we have:

$$\begin{aligned}\phi_{ik}^{(T)} &= 2\sum_{j=1}^{N} A_{ij}^{(T)} \frac{H_{jk}^{(T)}}{\sum_{k=1}^{r_T} H_{ik}^{(T)} H_{jk}^{(T)}} - 2\sum_{j=1}^{N} H_{jk}^{(T)} - 4\lambda \sum_{j=1}^{N} S_{ij} (\sum_{k=1}^{r_T} H_{ik}^{(T)} H_{jk}^{(T)}) H_{jk}^{(T)} \\ &\quad + 4\lambda \sum_{j=1}^{N} S_{ij} (\sum_{k=1}^{r_{T-1}} H_{ik}^{(T-1)} H_{jk}^{(T-1)}) H_{jk}^{(T)} - 2\beta H_{ik}^{(T)}. \end{aligned} \tag{8}$$

By the Karush-Kuhn-Tucker (KKT) conditions [5], $\phi_{ik}^{(t)} H_{ik}^{(t)} = 0$, so we could obtain the following equations for $H_{ik}^{(t)}$:

$$\begin{aligned} & H_{ik}^{(1)} \left(2\sum_{j=1}^{N} A_{ij}^{(1)} \frac{H_{jk}^{(1)}}{\sum_{k=1}^{r_1} H_{ik}^{(1)} H_{jk}^{(1)}} + 4\lambda \sum_{j=1}^{N} S_{ij} (\sum_{k=1}^{r_2} H_{ik}^{(2)} H_{jk}^{(2)}) H_{jk}^{(1)} \right) \\ = & H_{ik}^{(1)} \left(2\sum_{j=1}^{N} H_{jk}^{(1)} + 4\lambda \sum_{j=1}^{N} S_{ij} (\sum_{k=1}^{r_1} H_{ik}^{(1)} H_{jk}^{(1)}) H_{jk}^{(1)} + 2\beta H_{ik}^{(1)} \right), \end{aligned} \tag{9}$$

and for $t = 2, \ldots, T-1$:

$$\begin{aligned} & H_{ik}^{(t)} \left(2\sum_{j=1}^{N} A_{ij}^{(t)} \frac{H_{jk}^{(t)}}{\sum_{k=1}^{r_t} H_{ik}^{(t)} H_{jk}^{(t)}} + 4\lambda \sum_{j=1}^{N} S_{ij} (\sum_{k=1}^{r_{t+1}} H_{ik}^{(t+1)} H_{jk}^{(t+1)} + \sum_{k=1}^{r_{t-1}} H_{ik}^{(t-1)} H_{jk}^{(t-1)}) H_{jk}^{(t)} \right) \\ = & H_{ik}^{(t)} \left(2\sum_{j=1}^{N} H_{jk}^{(t)} + 8\lambda \sum_{j=1}^{N} S_{ij} (\sum_{k=1}^{r_t} H_{ik}^{(t)} H_{jk}^{(t)}) H_{jk}^{(t)} + 2\beta H_{ik}^{(t)} \right), \end{aligned} \tag{10}$$

and for $t = T$:

$$\begin{aligned} & H_{ik}^{(T)} \left(2\sum_{j=1}^{N} A_{ij}^{(T)} \frac{H_{jk}^{(T)}}{\sum_{k=1}^{r_T} H_{ik}^{(T)} H_{jk}^{(T)}} + 4\lambda \sum_{j=1}^{N} S_{ij} (\sum_{k=1}^{r_{T-1}} H_{ik}^{(T-1)} H_{jk}^{(T-1)}) H_{jk}^{(T)} \right) \\ = & H_{ik}^{(T)} \left(2\sum_{j=1}^{N} H_{jk}^{(T)} + 4\lambda \sum_{j=1}^{N} S_{ij} (\sum_{k=1}^{r_T} H_{ik}^{(T)} H_{jk}^{(T)}) H_{jk}^{(T)} + 2\beta H_{ik}^{(T)} \right). \end{aligned} \tag{11}$$

Through this rule, we obtain the following updating rule for $H^{(t)}$:

$$H_{ik}^{(1)} \leftarrow \frac{H_{ik}^{(1)}}{2} + \frac{1}{2} H_{ik}^{(1)} \frac{\sum_{j=1}^{N} \frac{A_{ij}}{\sum_{k=1}^{r_1} H_{ik}^{(1)} H_{jk}^{(1)}} H_{jk}^{(1)} + 2\lambda \sum_{j=1}^{N} S_{ij} (\sum_{k=1}^{r_2} H_{ik}^{(2)} H_{jk}^{(2)}) H_{jk}^{(1)}}{\sum_{j=1}^{N} H_{jk}^{(1)} + \beta H_{ik}^{(1)} + 2\lambda \sum_{j=1}^{N} S_{ij} (\sum_{k=1}^{r_1} H_{ik}^{(1)} H_{jk}^{(1)}) H_{jk}^{(1)}}, \tag{12}$$

for $t = 2, \ldots, T-1$,

$$H_{ik}^{(t)} \leftarrow \frac{H_{ik}^{(t)}}{2} + \frac{1}{2} H_{ik}^{(t)} \frac{\sum_{j=1}^{N} \frac{A_{ij}}{\sum_{k=1}^{r_t} H_{ik}^{(t)} H_{jk}^{(t)}} H_{jk}^{(t)} + 2\lambda \sum_{j=1}^{N} S_{ij} (\sum_{k=1}^{r_{t+1}} H_{ik}^{(t+1)} H_{jk}^{(t+1)} + \sum_{k=1}^{r_{t-1}} H_{ik}^{(t-1)} H_{jk}^{(t-1)}) H_{jk}^{(t)}}{\sum_{j=1}^{N} H_{jk}^{(t)} + \beta H_{ik}^{(t)} + 4\lambda \sum_{j=1}^{N} S_{ij} (\sum_{k=1}^{r_t} H_{ik}^{(t)} H_{jk}^{(t)}) H_{jk}^{(t)}}, \tag{13}$$

for $t = T$,

$$H_{ik}^{(T)} \leftarrow \frac{H_{ik}^{(T)}}{2} + \frac{1}{2} H_{ik}^{(T)} \frac{\sum_{j=1}^{N} \frac{A_{ij}}{\sum_{k=1}^{r_t} H_{ik}^{(T)} H_{jk}^{(T)}} H_{jk}^{(T)} + 2\lambda \sum_{j=1}^{N} S_{ij} (\sum_{k=1}^{r_{T-1}} H_{ik}^{(T-1)} H_{jk}^{(T-1)}) H_{jk}^{(T)}}{\sum_{j=1}^{N} H_{jk}^{(T)} + \beta H_{ik}^{(T)} + 2\lambda \sum_{j=1}^{N} S_{ij} (\sum_{k=1}^{r_T} H_{ik}^{(T)} H_{jk}^{(T)}) H_{jk}^{(T)}}. \tag{14}$$

Once each $H^{(t)}$ is initialized, we update $H^{(t)}$ according to Equations (12), (13) and (14) alternately until a stopping criterion is satisfied. Since the objective function in Equation (1) is non-convex, the final estimators of each $H^{(t)}$ depends on the initial

values. To reduce the risk of local minimization, we repeat the entire updating procedure 10 times with random initialization and choose the result that gives the lowest value of the objective function as the final estimator. In our implementation, the iteration process stops whenever $||H^{(1)}_{new} - H^{(1)}_{old}||_1 + ||H^{(T)}_{new} - H^{(T)}_{old}||_1 \leq 1e-6$. To avoid the case that this process converges too slowly, we also stop it if the number of iterations reaches 200. The procedure of identifying temporal protein complexes via our algorithm is described in Fig. S4.

3.2 Convergence analysis

We solve the optimization problem of TS-OCD via multiplicative updating rules which are special cases of gradient descent with an automatic step parameter selection. It could be proved that the objective function of our model is nonincreasing during each updating process and the iterative algorithm is guaranteed to find a least locally optimal solutions. Instead of proving this in theory, we validate the convergence experimentally. For each data set, we detect how the value of objective function changes with respect to the times of iterations. Fig. S8 shows the corresponding results on DIP and BioGrid with respect to the objective function of TS-OCD. From Fig. S8, we can find that the objective function of TS-OCD decrease sharply at the beginning and then change smoothly with respect to each update. When iterating the updating process for more than 200 times, the change of the objective function is small and can be neglected. Therefore, considering the problem of efficiency, we set the maximum iteration time to be 200.

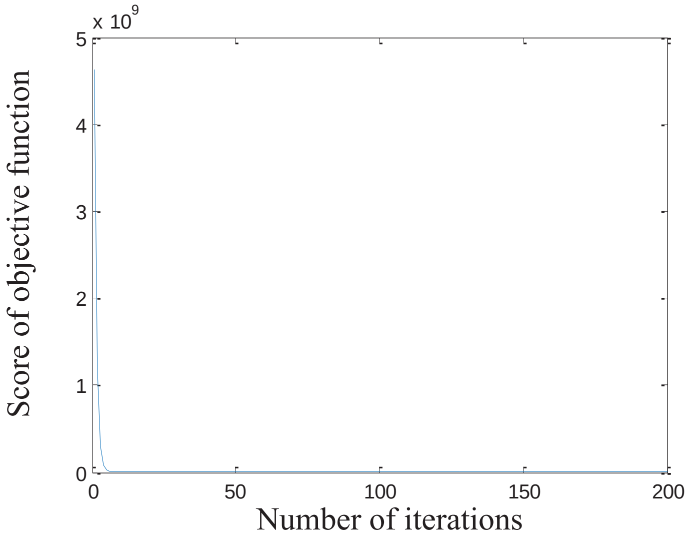

(a)

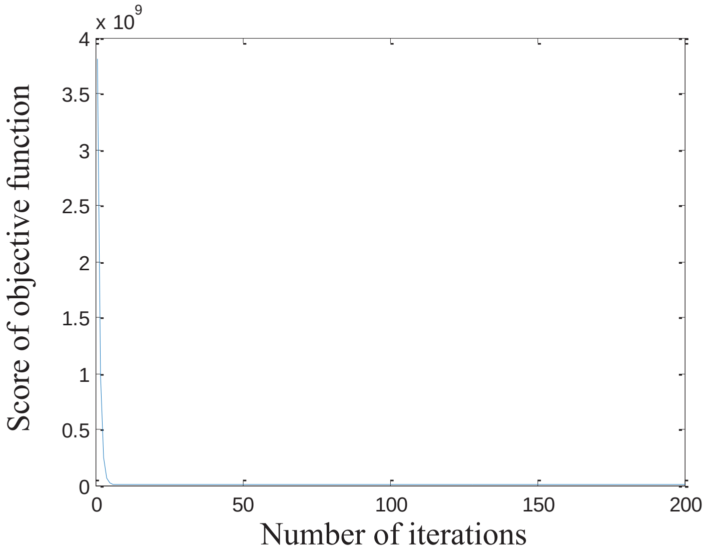

(b)

Figure S8: Convergence analysis of parameter estimation. For each figure, the x-axis denotes the number of iterations and the y-axis denotes the value of the objective function (1). (a) DIP. (b) BioGrid.

3.3 Data sets

We concentrate our study on yeast since it is a well studied model organism. The interactions derived from DIP [12] and BioGrid (version 3.1.77) [1] are used to test the performance respectively. We refer to them as DIP and BioGrid data sets. We download the BioGRID networks from the website of Nepusz *et al.*'s study (`http://membrane.cs.rhul.ac.uk/static/cl1/cl1_datasets.zip`) [9]. To construct dynamic PPI networks, we integrate time-course gene expression data with physical PPI networks. The gene expression data are download from Gene Expression Omnibus (GEO) [2] with the accession number GSE3431 and we only use the 3552 significantly periodic genes [15]. Among the 3552 genes, 2389 occur in DIP and 3057 occur in BioGrid. Thus, we retain these genes and the corresponding interactions among them in DIP and BioGrid respectively. Table S4 lists several topological features of the two networks and shows that they have different structural characterizations. The topological differences between them can be used to test the generalization of each considered approach.

Table S4: Statistics of topological features of the used networks.

	BioGrid	DIP
Number of proteins	3057	2389
Number of interactions	19727	7044
Average number of neighbors	12.906	5.897
Centralization	0.243	0.058
Clustering coefficient	0.201	0.093
Number of connected components	2	35
Density	0.004	0.002
Diameter	8	12

These statistics are calculated using software Cytoscape [13].

We use the CYC2008 [10] and MIPS [8] benchmarks as the gold standards of yeast protein complexes. The CYC2008 catalogue is downloaded from `http://wodaklab.org/cyc2008/downloads` on April 6, 2013. For the MIPS gold standard, we use the dataset which has been used in [9] and can be download from `http://membrane.cs.rhul.ac.uk/static/cl1/cl1$_$gold_standard.zip`. For details of the construction of this benchmark, please refer to [9]. We map both the two reference sets onto each PPI network and filter them based on size in a similar manner of [9] (`http://membrane.cs.rhul.ac.uk/static/cl1/additional_information.html`). The two gold standards are used independently for evaluation of the methods. The general properties of the reference sets are listed in Table S5.

Table S5: Statistics of the gold standard complexes we use.

		All	DIP	BioGrid
CYC2008	Number of complexes	408	103	116
	Number of proteins	1,627	430	580
	Number of proteins in ≥ 2 complexes	211	55	60
MIPS	Number of complexes	203	136	146
	Number of proteins	1,189	524	686
	Number of proteins in ≥ 2 complexes	820	397	456

Here "All" denotes the statistics of each reference set which is not mapped onto the PPI network and filtered in terms of size.

3.4 Evaluation metrics

To evaluate the performance for complex detection, two independent quality criteria—PR metric [14] and f-measure [7], are used to assess the similarity between the predicted complexes and the known complexes. These two metrics have complementary strengths, so they could evaluate the performance from different perspectives. Between these two measures, PR metric could judge how well the predicted complexes correspond to known complexes by considering the number of proteins in each complex as well as the overlaps between predicted complex and know complexes. While f-measure assess the performance from a macro perspective (Recall measures what fraction of the known complexes are matched by the predicted complexes, and Precision measures what fraction of the predicted complexes are matched with known complexes).

We first give some notations before describing these measures. Let P denote the number of complexes detected by a particular algorithm and T denote the number of reference complexes. Let C_i represents the set of proteins belong to the i-th detected complex and G_j represents the set of proteins belong to the j-th reference complex.

We say a detected complex C_i and a reference complex G_j match each other if:

$$\frac{|C_i \bigcap G_j|^2}{|C_i||G_j|} > \nu. \tag{15}$$

where ν is an input parameter between 0 and 1 which is usually set to 0.25 [7]. Therefore, in this study, we fix $\nu = 0.25$. Given a set of predicted complexes $\mathcal{C} = \{C_1, C_2, \cdots, C_P\}$ and a set of reference complexes $\mathcal{G} = \{G_1, G_2, \cdots, G_Q\}$,

Precision and Recall are defined as follows:

$$Precision = \frac{|\{C_i|C_i \in \mathcal{C} \wedge \exists G_j \in \mathcal{G}, G_j \ matches \ C_i\}|}{P}, \tag{16}$$

$$Recall = \frac{|\{G_j|G_j \in \mathcal{G} \wedge \exists C_i \in \mathcal{C}, C_i \ matches \ G_j\}|}{Q}. \tag{17}$$

In order to take into account of both the Precision and Recall, an integrated method called f-measure is used.

$$f - measure = \frac{2 \times Precision \times Recall}{Precision + Recall}. \tag{18}$$

The other measure is defined as follows:

PR measure: The precision-recall (PR)-based score $PR_{i,j}$ between a predicted complex C_i and a reference complex G_j is calculated by $PR_{i,j} = \frac{|C_i \bigcap G_j|}{|C_i|} \times \frac{|C_i \bigcap G_j|}{|G_j|}$. The first part $\frac{|C_i \bigcap G_j|}{|C_i|}$ is the precision metric which measures what fraction of the proteins in predicted complex C_i correspond to reference complex G_j, and the second part $\frac{|C_i \bigcap G_j|}{|G_j|}$ is the recall metric which measures how much of reference complex G_j is recovered by predicted complex C_i. For each predicted complex C_i, we find the reference complex that maximizes the PR score between them, which is defined as $PRC_i = \max_j PR_{i,j}$ and for each reference complex G_j, we try to find the predicted complex that maximizes the PR score between them, that is $PRG_j = \max_i PR_{i,j}$ for the ***PR*** measure.

Taking average over all the predicted complexes, weighted by the size of each predicted complex, we obtain PRC as follows:

$$PRC = \frac{\sum_{i=1}^{P} |C_i| \cdot PRC_i}{\sum_{i=1}^{P} |C_i|}. \tag{19}$$

Similarly, the measures PRG for the T reference complexes is $PRG = \frac{\sum_{j=1}^{Q} |G_j| \cdot PRG_j}{\sum_{j=1}^{Q} |G_j|}$. Finally, we use PR which is the harmonic mean of PRC and PRG to quantify the accuracy of the predicted complexes:

$$PR = \frac{2 \times PRC \times PRG}{PRC + PRG}. \tag{20}$$

. We implement the Matlab code for the calculation of the PR score according to the formulations described in [14].

3.5 Effect of random restart

Since the objective function of TS-OCD is not convex, we can not guarantee the multiplicative updating rule-based iterative algorithm will converge to the global minimum. To avoid local minimization, we repeat the entire calculation 10 times with random restarts and choose the result that gives the lowest value of the objective function. We limit the number of repetitions to be 10 because of the time cost of each repetitions. As a result, we can not guarantee the final estimator is the globally optimum solution and the result is not deterministic. We therefore focus on the variability of the results with random restarts. We repeat the entire procedure 10 times with random restarts and see how the results are affected by different restarts. For DIP and BioGrid, the corresponding results are shown in Fig. S9 and S10 respectively.

From Fig. S9 and S10, we can find that, with different random restarts, the performance of TS-OCD change obviously. However, within ten random restarts, we could obtain reasonable good results. Therefore, in this study, we repeat the entire calculation 10 times with random restarts and choose the result that gives the lowest value of the objective function. Note better results will be obtained if more repetitions are conducted.

3.6 Effect of smooth regularization

To investigate the benefits of using the smooth regularization, we compare the performance of our model with and without smooth regularization (denoted as NS-OCD, Non-Smooth Overlapping Complex Detection). We apply TS-OCD and NS-OCD on DIP and BioGrid dynamic networks respectively and evaluate their performance in terms of two metrics (PR and f-measure) based on two gold standards (CYC2008 and MIPS). For NS-OCD, we also fix the value of β to be 2^4. Fig. S1 and Fig. S2 shows the comparative performance of TS-OCD and NS-OCD on DIP and BioGrid dynamic networks using the benchmark CYC2008 and MIPS.

From Fig. S1 and Fig. S2, we can find that TS-OCD performs better than NS-OCD on both DIP and BioGrid data. For instance, on BioGrid data, the f-measure for TS-OCD and NS-OCD are 0.487 and 0.436 respectively with respect to CYC2008. That is, the complexes detected by TS-OCD have better quality than those detected by NS-OCD. In most cases, the living system is more likely to change gradually other than dramatically. Therefore, with time smooth regularization, the TS-OCD model may help to better capture the temporal behaviors of protein complexes.

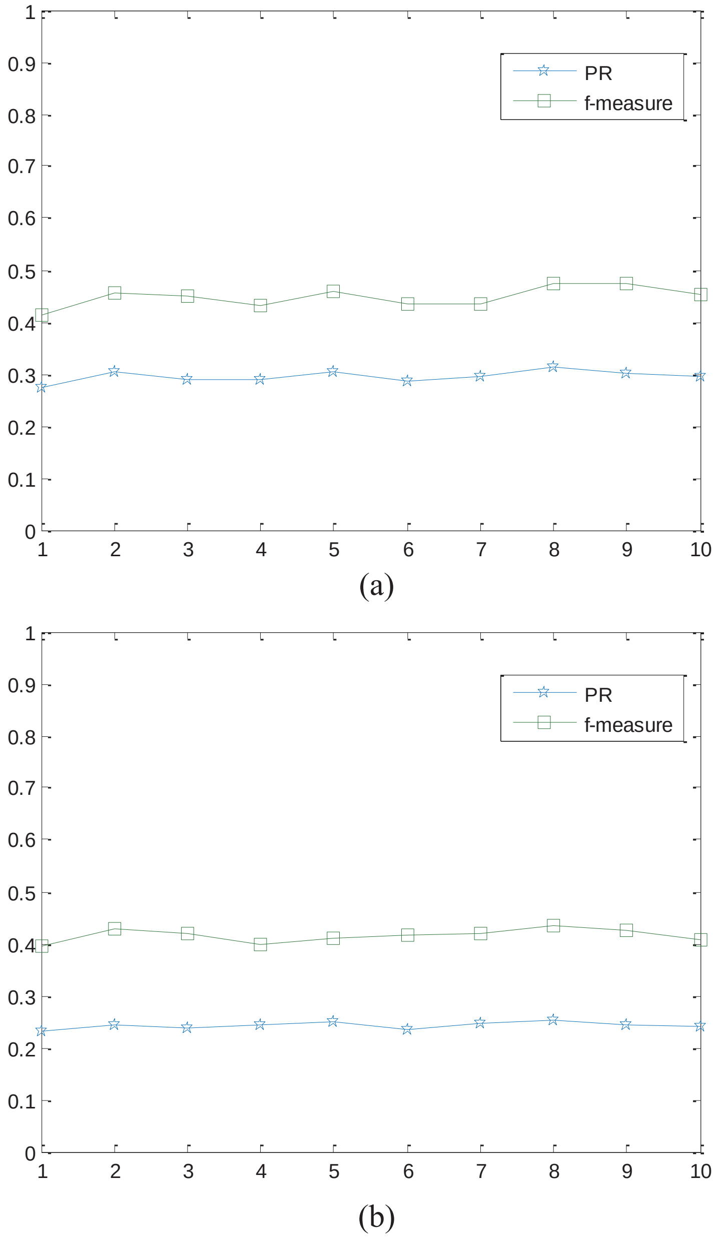

Figure S9: Performance of TS-OCD with different random restarts in terms of two metrics with respect to (a) CYC2008 and (b) MIPS on DIP.

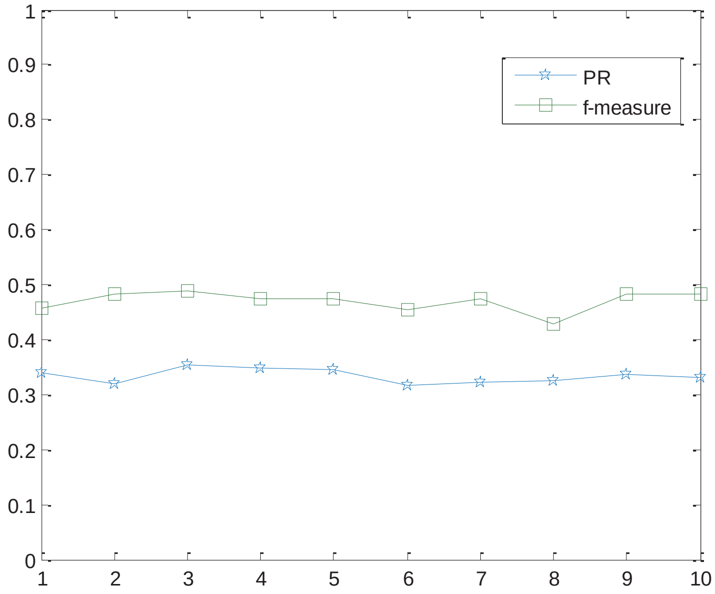

(a)

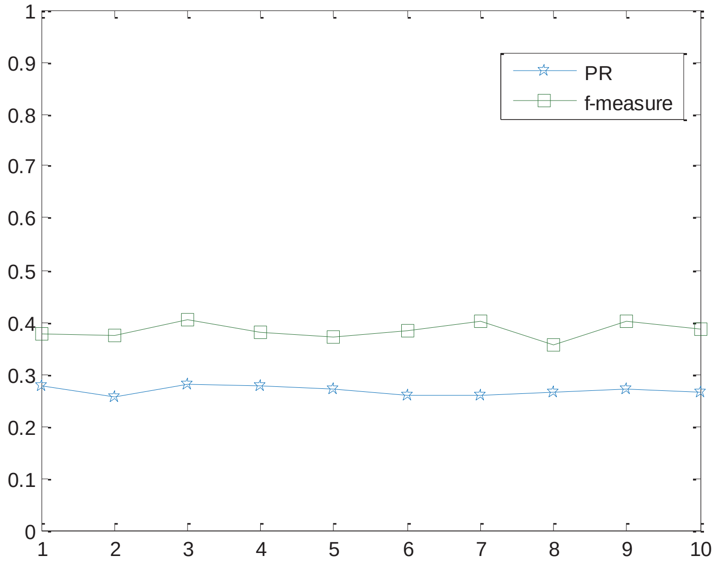

(b)

Figure S10: Performance of TS-OCD with different random restarts in terms of two metrics with respect to (a) CYC2008 and (b) MIPS on BioGrid.

Table S6: Characteristics of the compared algorithms

Algorithm	Downloading website	Version
ClusterONE	`http://www.paccanarolab.org/cluster-one/index.html`	0.94
COACH	`http://www1.i2r.a-star.edu.sg/~xlli/`	-
MCL	`http://micans.org/mcl/`	09-308
MINE	`http://www.biomedcentral.com/1471-2105/12/192`	1.5
SPICi	`http://compbio.cs.princeton.edu/spici/`	-

Table S7: Parameters selected for COACH

Network	Static network		Dynamic network	
	DIP	BioGrid	DIP	BioGrid
ω	0.05	0	0	0

3.7 Parameter settings of compared algorithms

In this paper, in order to evaluate the performance of our method in detecting protein complexes, we compare it with five existing methods: ClusterONE [9], COACH [16], MCL [3], MINE [11], and SPICi [4]. Table S6 lists the websites where we download the softwares of these algorithms and the version numbers of these softwares. Before describing the parameter settings for each algorithm, we declare several general consideration first. Since the performance of each algorithm depends on the choice of its inherent parameters and the data set under consideration, for all the considered algorithms, we optimize the parameters that yield the best results. To avoid evaluation bias, we also consider the following three criterions:

- Two quality metrics (PR score and f-measure) are used to evaluate the performance of each algorithm.
- Two different gold standards (the MIPS complexes and the CYC2008 complexes) are used.
- For each algorithm, the final results are obtained by choosing the parameters that yield the best performance which are measured by the f-measure on the MIPS complexes.

We briefly review the main features of these algorithms and the setting of parameters for each algorithm in the following text.

ClusterONE

ClusterONE is recently proposed by Nepusz *et al.* [9] to detect overlapping protein complexes in PPI networks based on overlapping neighborhood expansion. As suggested by the authors, we do not tune the parameters for a particular network. Thus, we use the default settings of parameters in the software.

COACH

COACH, as a core-attachment based method, has the following two steps to detect protein complexes from PPI networks. First, it detects local dense clusters as cores. Second, cores will be expanded to complexes by including attachment proteins that are closely connected to cores. There is a parameter ω in the first step to control the overlap between identified cores, e.g., a higher ω allow more common proteins between two different cores. In this study, we try different values of ω, ranges from 0 to 0.2 with 0.05 increment. The optimal value of ω for each PPI network is shown in Table S7.

MCL

Markov Clustering Algorithm (MCL) [3] is a competing protein complex detection algorithm and has been developed in different languages, such as JAVA, R and C. The key parameter of MCL is inflation, which tunes the granularity of clustering. Here, we try different values of inflation, ranges from 1.2 to 5.0 with 0.2 increment. The optimal value of inflation for each PPI network is shown in Table S8.

Table S8: Parameters selected for MCL

Network	Static network		Dynamic network	
	DIP	BioGrid	DIP	BioGrid
Inflation	2.2	2.6	2.6	2.2

Table S9: Parameters selected for MINE

Network	Static network		Dynamic network	
	DIP	BioGrid	DIP	BioGrid
node score cutoff	0.1	0.1	0.1	0.1
modularity score cutoff	0.3	0.2	0.3	0.2
depth limit	3	3	3	3

Table S10: Parameters selected for SPICi

Network	Static network		Dynamic network	
	DIP	BioGrid	DIP	BioGrid
density	0.8	0.7	0.8	0.7

MINE

MINE [11] can identify highly modular sets of proteins within highly interconnected PPI networks. The key parameters of MINE are node score cutoff and modularity score cutoff. We try different value of node score cutoff and modularity score cutoff (from 0.1 to 1 with 0.1 as the step size) and 3 settings of depth limit (3, 4, 5). For the other parameters, without stating, we use the default values in the software. The optimal values of the parameters of MINE for each PPI network are listed in Table S9.

SPICi

SPICi [4] is a computationally efficient local network clustering algorithm for large biological networks, which can be applied on PPI networks for complex detection. SPICi has two parameters: the density threshold and the support threshold. Here, we try different values of density threshold, ranges from 0.1 to 1 with 0.1 increment. For the other parameters, we use the default settings in the software. Table S10 lists the optimal value of density parameter for each PPI networks.

References

[1] Andrew Chatr-aryamontri et al. The biogrid interaction database: 2013 update. *Nucleic Acids Res.*, 41(D1):D816–D823, 2013.

[2] Ron Edgar, Michael Domrachev, and Alex E Lash. Gene expression omnibus: Ncbi gene expression and hybridization array data repository. *Nucleic acids research*, 30(1):207–210, 2002.

[3] A.J. Enright et al. An efficient algorithm for large-scale detection of protein families. *Nucleic Acids Res.*, 30(7):1575–1584, 2002.

[4] Peng Jiang and Mona Singh. Spici: a fast clustering algorithm for large biological networks. *Bioinformatics*, 26(8):1105 – 1111, 2010.

[5] H.W. Kuhn and A.W. Tucker. Nonlinear programming. In *Proceedings of the second Berkeley symposium on mathematical statistics and probability*, volume 1, pp. 481–492. California, 1951.

[6] Daniel D. Lee and H. Sebastian Seung. Algorithms for non-negative matrix factorization. In *Advances in neural information processing systems*, volume 13, pp. 556–562, 2001.

[7] Xiaoli Li et al. Computational approaches for detecting protein complexes from protein interaction networks: a survey. *BMC Genomics*, 11(Suppl 1):S3, 2010.

[8] H.W. Mewes et al. Mips: analysis and annotation of proteins from whole genomes. *Nucleic Acids Res.*, 32(suppl 1):D41–D44, 2004.

[9] T. Nepusz et al. Detecting overlapping protein complexes in protein-protein interaction networks. *Nat. Methods*, 9(5):471–472, 2012.

[10] Shuye Pu et al. Up-to-date catalogues of yeast protein complexes. *Nucleic Acids Res.*, 37(3):825–831, 2009.

[11] Kahn Rhrissorrakrai and Kristin C Gunsalus. Mine: module identification in networks. *BMC Bioinformatics*, 12(1):192, 2011.

[12] Lukasz Salwinski et al. The database of interacting proteins: 2004 update. *Nucleic Acids Res.*, 32(suppl 1):D449–D451, 2004.

[13] Michael E Smoot et al. Cytoscape 2.8: new features for data integration and network visualization. *Bioinformatics*, 27(3):431–432, 2011.

[14] J. Song and M. Singh. How and when should interactome-derived clusters be used to predict functional modules and protein function? *Bioinformatics*, 25(23):3143–3150, 2009.

[15] Benjamin P Tu, Andrzej Kudlicki, Maga Rowicka, and Steven L McKnight. Logic of the yeast metabolic cycle: temporal compartmentalization of cellular processes. *Science*, 310(5751):1152–1158, 2005.

[16] Min Wu, Xiaoli Li, Chee-Keong Kwoh, and See-Kiong Ng. A core-attachment based method to detect protein complexes in ppi networks. *BMC bioinformatics*, 10(1):169, 2009.
